# Multi-Omics and Machine Learning-Based Characterization of the Lactylation Microenvironment and Biomarker Identification in Crohn’s Disease Intestinal Fibrosis

**DOI:** 10.3390/ijms27146343

**Published:** 2026-07-17

**Authors:** Qi Sun, Xing-Yu Cui, Jun-Kun Zhan

**Affiliations:** 1 Department of General Surgery, The Second Xiangya Hospital, Central South University, Changsha 410011, China; 2 Institute of Aging and Age-Related Disease Research, Central South University, Changsha 410011, China; 3 Department of Geriatrics, The Second Xiangya Hospital, Central South University, 139 Renmin Middle Road, Furong District, Changsha 410011, China

**Keywords:** Crohn’s disease, intestinal fibrosis, lactylation, machine learning, single-cell RNA-seq, spatial transcriptomics

## Abstract

Crohn’s disease (CD) is characterized by transmural inflammation and intestinal fibrosis, in which metabolic reprogramming may contribute to fibrotic remodeling through lactate-associated epigenetic and transcriptional regulation, but key cellular states and candidate biomarkers remain unclear. Therefore, we integrated single-cell RNA sequencing (scRNA-seq) with a lactylation-associated transcriptional score to estimate lactate/lactylation-related transcriptional activity in CD intestinal tissues. High-dimensional weighted gene co-expression network analysis (hdWGCNA) and an integrated machine learning framework identified core lactylation-associated biomarkers, which were validated in clinical tissue and a TNBS-induced mouse model. Additionally, we evaluated the ability of these core genes to predict anti-TNF-α treatment response in an independent clinical cohort, and analyzed cellular communication and trajectories, using CellChat, Monocle 2, and spatial transcriptomics. In this study, an enterocyte state with high lactylation-associated transcriptional scores was identified. *CALD1* and *CALM1* were identified as core transcriptional biomarkers associated with this high-lactylation-score epithelial state. A diagnostic nomogram exhibited robust predictive performance (AUC > 0.75, *p* < 0.05). Notably, increased *CALM1* expression was associated with favorable anti-TNF-α response (AUC > 0.70, *p* < 0.05). Mechanistically, *CALD1*/*CALM1* dysregulation was associated with enhanced *GDF15*- and *MIF*-related signaling, potentially contributing to endothelial and macrophage-associated microenvironmental remodeling. Together, *CALD1* and *CALM1* represent lactylation-score-associated transcriptional biomarkers that may link metabolic reprogramming, epithelial dysfunction, and fibrotic remodeling in CD, serving as candidate diagnostic and treatment-response-associated biomarkers.

## 1. Introduction

Crohn’s disease (CD) is a major subtype of inflammatory bowel disease (IBD). Its core features are chronic, recurrent, and transmural intestinal inflammation that can affect the entire digestive tract [1,2]. As the disease progresses, repeated cycles of inflammatory injury and repair lead to excessive extracellular matrix (ECM) deposition, ultimately causing intestinal wall thickening, stiffening, and luminal strictures. These irreversible structural changes are the leading causes of recurrent hospitalizations and surgical resections in patients [3]. Biologic therapies, particularly anti-TNF-α monoclonal antibodies, have substantially improved inflammatory control and mucosal healing in CD. However, these agents primarily target inflammatory pathways and do not directly reverse established intestinal fibrosis. As a result, fibrostenotic complications, including intestinal strictures and obstruction, remain major clinical challenges, and endoscopic dilation or surgical resection is still required in a substantial proportion of patients with established fibrotic disease [4,5]. This clinical dilemma highlights the “inflammation–fibrosis decoupling” phenomenon. Once initiated, intestinal fibrosis may establish self-sustaining molecular mechanisms independent of acute inflammation. Even when local inflammation is effectively controlled, the fibrotic remodeling process can continue to progress [6]. Therefore, elucidating the core driving mechanisms of intestinal fibrosis, and identifying early diagnostic biomarkers and predictive targets for treatment efficacy, are urgent priorities in the current IBD field.

Emerging evidence suggests that metabolic reprogramming within the tissue microenvironment is a critical driver of intestinal fibrotic process [7,8]. In the microenvironments of chronic inflammation and fibrotic tissues, hypoxia, nutrient deprivation, and metabolic waste accumulation are common pathological features. To adapt to this stress, cells undergo significant metabolic reprogramming. Rather than referring to aerobic glycolysis in a strict sense, these pathological conditions are more accurately described as being associated with enhanced glycolytic metabolism or Warburg-like glycolytic reprogramming. Such metabolic adaptation enables cells to rapidly generate energy and acquire biosynthetic precursors under inflammatory, hypoxic, and metabolically stressed conditions. However, this metabolic shift also causes a massive accumulation of the byproduct lactate [9,10]. Rather than serving merely as a glycolytic end-product, lactate has been reported to function as a bioactive metabolic signal involved in chronic inflammation and tissue remodeling [11]. A lactate-rich milieu may contribute to fibrotic progression through several interconnected mechanisms. First, extracellular lactate accumulation can promote local acidification and facilitate activation of latent TGF-β signaling [12], thereby enhancing fibroblast-to-myofibroblast differentiation and extracellular matrix deposition. Second, lactate may act as a paracrine metabolic signal that modulates macrophage polarization, inflammatory-cell recruitment, angiogenesis, vascular remodeling, and stromal-cell activation, all of which are closely associated with chronic tissue repair and fibrosis. Third, intracellular lactate can serve as a substrate for lysine lactylation, particularly histone lactylation, thereby linking glycolytic metabolic stress to sustained transcriptional activation of inflammation- and fibrosis-related gene programs [13,14]. Specifically in the intestinal inflammatory microenvironment, key cell populations such as macrophages, T cells, and intestinal stromal cells undergo this precise metabolic adaptation, releasing abundant lactate. Together, they construct a lactate-rich “metabo-inflammatory microenvironment” that may provide the necessary conditions for the pathogenesis and fibrotic progression of CD [15,16].

**Figure 1:**
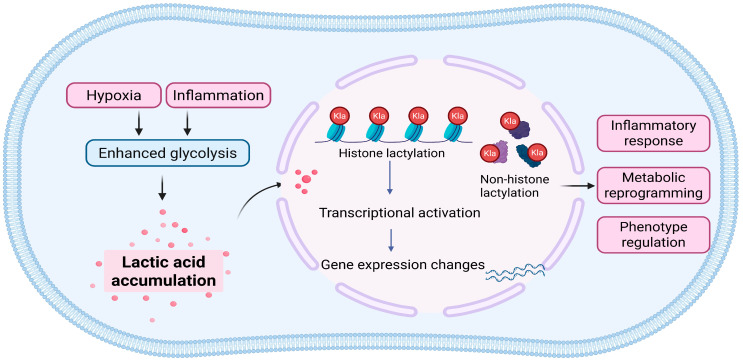
Overview of the molecular mechanism and biological effects of lactylation. Hypoxia and inflammation promote glycolytic activation and lactate accumulation. Elevated lactate levels are associated with histone and non-histone lactylation. Histone lactylation induces transcriptional activation and gene expression changes, while non-histone lactylation may regulate protein-related signaling events. These processes collectively contribute to inflammatory response, metabolic reprogramming, and phenotype regulation.

Although lactate accumulation plays a critical role in driving fibrosis, how this specific metabolite translates into persistent gene expression changes or cellular functional remodeling remains unclear. The recent discovery of lactate-derived histone lysine lactylation provides a mechanistic link between lactate accumulation and persistent transcriptional remodeling. As a metabolic–epigenetic modification, histone lactylation uses lactate-derived lactyl groups to modify histone lysine residues, thereby regulating chromatin accessibility and gene transcription. Emerging evidence also suggests that non-histone lactylation may further modulate cellular function at the post-translational level [10,14]. Currently, this modification mechanism has been shown to participate in processes such as M1/M2 polarization switching and tumor immune evasion [17]. Macrophage polarization is a representative example of lactylation-mediated immune remodeling. Rather than following a strict M1/M2 dichotomy, macrophages in vivo display dynamic functional states shaped by inflammatory and metabolic cues [18,19]. Under chronically inflamed conditions, lactate accumulation can influence macrophage functional polarization [20], while lactate-derived histone lactylation may regulate macrophage transcriptional programs, including inflammatory, reparative, and wound-healing responses [14]. These findings suggest that lactylation may link metabolic stress to macrophage functional remodeling, chronic inflammation, tissue repair, and fibrotic remodeling in CD [21]. In fibrotic diseases, studies have demonstrated that histone lactylation promotes the expression of fibrosis-related genes in the lung, liver, and kidney [10,22]. These lactate-centered mechanisms, including glycolytic activation, lactate accumulation, histone/non-histone lactylation, transcriptional remodeling, and downstream inflammatory or phenotypic regulation, are summarized in Figure 1. However, during the progression of CD intestinal fibrosis, the specific cellular carriers of lactylation modification, the core target gene network, and whether lactylation-related transcriptional remodeling contributes to the limited ability of anti-TNF-α therapy to reverse established fibrosis, urgently require further elucidation.

Therefore, this study aims to explore the lactylation-related molecular and cellular features associated with intestinal fibrosis in CD through a multi-omics integrative analysis. We integrated single-cell transcriptomics, high-dimensional weighted gene co-expression network analysis (hdWGCNA), ensemble machine learning, spatial transcriptomics, and multiple independent cohort validations to characterize cell subpopulations with relatively elevated lactylation-associated transcriptional activity and to screen candidate lactylation-associated biomarkers at a single-cell resolution. Furthermore, we seek to investigate potential microenvironmental communication patterns involving these cell populations and provide candidate biomarkers or targets for further evaluation in the diagnosis and management of CD-associated intestinal fibrosis.

## 2. Results

The Results Section is organized into four sequential modules. First, we used scRNA-seq data to define the intestinal cellular landscape of CD and identify cell populations with relatively elevated lactylation-associated transcriptional scores. Second, we focused on the High_Enterocytes state and applied hdWGCNA and machine learning analyses to prioritize lactylation-score-associated candidate genes. Third, we validated the core genes in independent transcriptomic cohorts, clinical intestinal tissues, and a TNBS-induced murine colitis model. Finally, we explored the potential microenvironmental relevance of these genes using anti-TNF-α response analysis, CellChat-based cell communication, pseudotime analysis, and spatial transcriptomic mapping.

### 2.1. Single-Cell Landscape and Lactylation-Associated Transcriptional Scoring in CD Intestine

**Figure 2:**
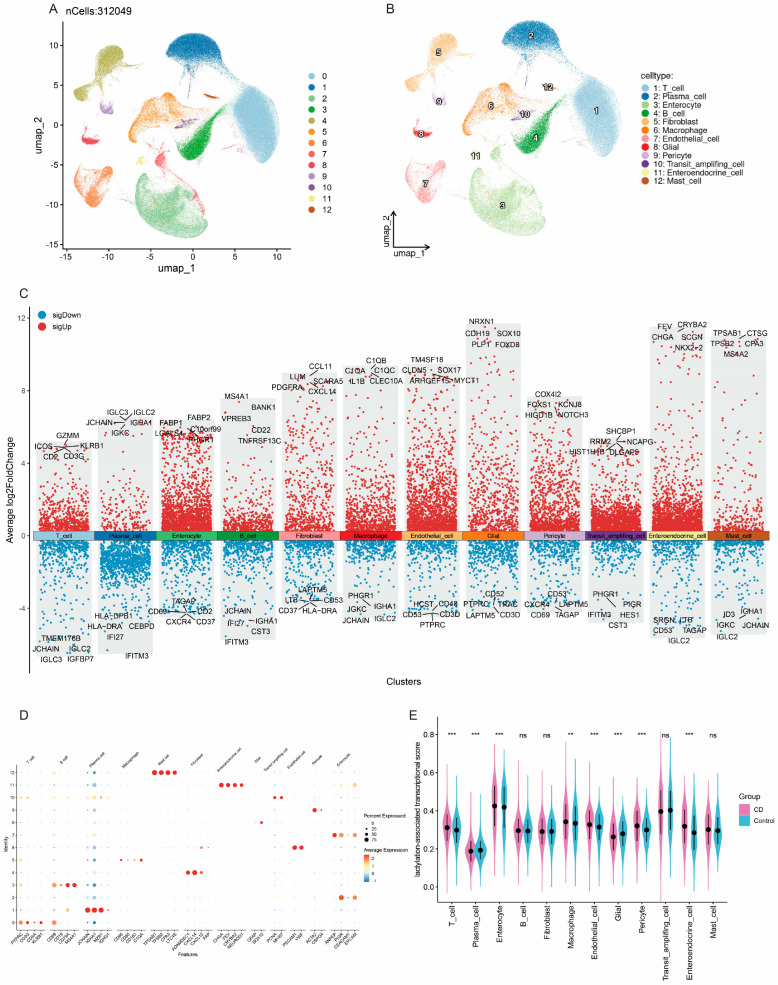
Single-cell transcriptomic landscape of the Crohn’s disease intestine and cell type identification. (**A**) UMAP visualization illustrating the distribution of cellular clusters (*n* = 312,049 cells, comprising 13 major clusters). (**B**) Annotation map of the 12 major cell types based on canonical marker genes. (**C**) Volcano plots highlighting the cell-type-specific highly expressed genes. (**D**) Dot plot displaying the expression patterns of representative marker genes across the identified cell types. (**E**) Violin plots comparing the discrepancies in lactylation scores across various cell types between the CD and healthy control groups. Panel (**E**): Wilcoxon rank-sum test with BH correction. ns, not significant; ** *p* < 0.01; *** *p* < 0.001.

To characterize the cellular landscape of the CD intestinal microenvironment and to identify cell populations potentially associated with lactylation-related transcriptional remodeling, we analyzed the GSE282122 scRNA-seq dataset as the primary single-cell discovery cohort. Following rigorous quality control, a total of 312,049 high-quality single-cell profiles were retained (Supplementary Figure S1A,B). Based on UMAP dimensionality reduction, these cells were partitioned into 13 distinct clusters (Supplementary Figure S1C) and subsequently annotated into 12 major cell types utilizing canonical marker genes, including epithelial, immune, stromal, and vascular-associated populations (Figure 2A–D).

Subsequently, leveraging a predefined set of lactate metabolism- and lactylation-related genes, the lactylation-associated transcriptional score of each individual cell was calculated using the AddModuleScore function in Seurat. This score was interpreted as the relative enrichment of lactylation-related transcriptional activity at the single-cell level, rather than as a direct measurement of global or site-specific protein lactylation. The results indicated altered lactylation-associated transcriptional scores across multiple cell types in the intestinal tissues of CD patients compared with healthy controls (Figure 2E). Enterocytes exhibited relatively high lactylation-associated transcriptional scores in CD samples, whereas healthy control enterocytes also showed a relatively high baseline score. This pattern suggested that lactylation-associated transcriptional activity may be relatively prominent within the enterocyte compartment and appeared to become more heterogeneous under CD conditions. Within CD enterocytes, a subset of cells displayed relatively higher lactylation-associated transcriptional scores than the remaining enterocytes; this transcriptionally defined enterocyte state was designated as “High_Enterocytes”. These observations prompted us to further examine the transcriptional features of this high-lactylation-score epithelial state.

### 2.2. Identification of Gene Co-Expression Modules Associated with the High_Enterocytes State by HdWGCNA

**Figure 3:**
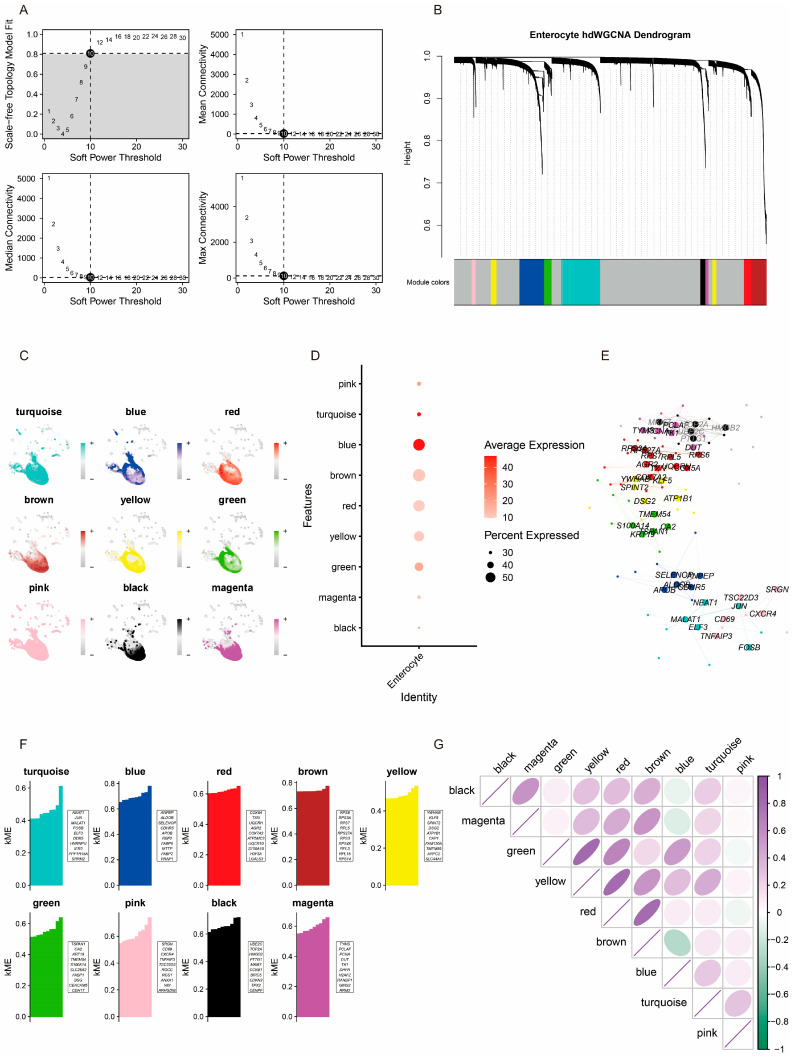
The hdWGCNA of enterocytes and identification of critical co-expression modules. (**A**) Network topology analysis and the selection of the soft-thresholding power. The gray shaded region represents scale-free topology fit values below the predefined threshold of *R*^2^ = 0.8. The horizontal dashed line indicates the *R*^2^ = 0.8 threshold, and the vertical dashed lines indicate the selected soft-thresholding power of β = 10. (**B**) Hierarchical clustering dendrogram of the gene co-expression modules, the colored bands beneath the dendrogram indicate the gene co-expression modules assigned by hdWGCNA, with each color representing a distinct modul. (**C**) UMAP visualization of the MEs. (**D**) Dot plot illustrating the expression patterns of the co-expression modules across enterocytes. (**E**) Co-expression network of hub genes within the critical modules (node colors denote their corresponding modules). (**F**) Distribution of kME values for the top-ranking hub genes within each module. (**G**) Heatmap illustrating the correlations among the module eigengenes. In panel (**A**), β = 10 was selected for enterocyte hdWGCNA based on the scale-free topology model fit index (R^2^ = 0.809).

Given the observed heterogeneity within the enterocyte compartment, we next performed hdWGCNA to explore gene co-expression programs associated with the High_Enterocytes state. This analysis aimed to determine which epithelial transcriptional modules were linked to the high-lactylation-score enterocyte state and CD status. Following network topology analysis, a soft thresholding power of 10 was selected to construct a scale-free co-expression network (Figure 3A). At β = 10, the scale-free topology model fit index was R^2^ = 0.809, meeting the predefined scale-free topology fit criterion of 0.8. Based on the TOM and hierarchical clustering, multiple gene co-expression modules with distinct expression patterns were identified (Figure 3B,D). The UMAP of the MEs illustrated the distribution characteristics of each module across various cellular states (Figure 3C).

Module–trait correlation analysis (Figure 3D) revealed that the blue module exhibited the most robust positive correlation with both the High_Enterocytes state and the CD state. Therefore, this module was selected as the key epithelial module for downstream candidate-gene prioritization. Based on kME values, representative hub genes within the major modules were further examined (Figure 3E,F). The blue module contained genes related to epithelial metabolism, nutrient absorption, and intestinal epithelial function, including *ANPEP*, *ALDOB*, and *APOB*. The pink module included inflammation- and immune activation-related genes, such as *SRGN*, *CD69*, and *ANXA1*. In addition, the black module contained cell-cycle- and proliferation-related genes, including *TOP2A* and *MKI67*. Module correlation analysis further indicated the potential interconnections among these functional modules (Figure 3G). These results suggest that High_Enterocytes may be characterized by transcriptional programs related to epithelial metabolic remodeling, inflammatory activation, and proliferative or repair-associated responses. The blue module was therefore used as the principal High_Enterocytes-associated module for subsequent lactylation-related candidate-gene screening.

### 2.3. Identification of Core Lactylation-Score-Associated Candidate Genes Using Multi-Algorithm Machine Learning

**Figure 4:**
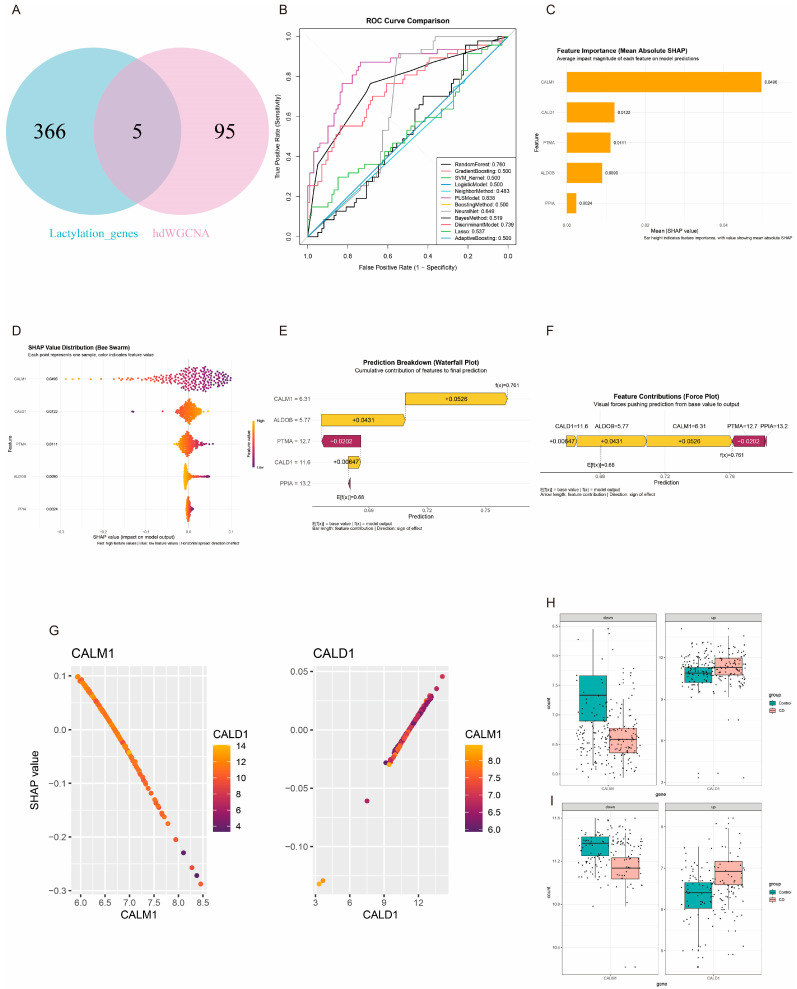
Identification of core lactylation-related genes in Crohn’s disease based on multi-algorithm machine learning and the SHAP model. (**A**) Venn diagram illustrating the intersection between the Lactylation_genes and the hub genes from the critical hdWGCNA module (yielding five candidate genes). (**B**) Comparison of ROC curves evaluating the predictive performance across the 12 machine learning algorithms, the gray dotted diagonal line represents the no-discrimination reference line corresponding to an AUC of 0.5. (**C**) Feature importance ranking based on the Random Forest model (evaluated by mean absolute SHAP values). (**D**) SHAP summary plot (beeswarm plot) of the top five features, demonstrating the nonlinear impact of feature distributions on the model output. (**E**,**F**) Local explanation plots detailing the prediction results for individual samples. (**G**) SHAP dependence plots illustrating the relationship between core gene expression levels and their corresponding SHAP values. (**H**,**I**) Differential expression levels of the core genes *CALD1* and *CALM1* in the training cohort (**H**) and the independent validation cohort (**I**). Panels (**H**,**I**): Mann–Whitney U test.

To further screen for core genes harboring potential clinical diagnostic and biological regulatory value, we established a multi-algorithm machine learning pipeline. By intersecting the predefined lactate metabolism- and lactylation-related gene set with the top 100 hub genes from the blue module, a total of five overlapping genes (*CALM1*, *CALD1*, *PTMA*, *ALDOB*, and *PPIA*) were acquired as lactylation-related candidate feature genes (Figure 4A,C). These five genes were defined as lactylation-related candidate feature genes based on their overlap with the predefined gene set and High_Enterocytes-associated module genes, which refers to transcriptional-signature-level relevance rather than direct site-specific protein lactylation validation.

Using the expression matrix of these five candidate genes in the GSE186582 training cohort, we then constructed and compared 12 machine learning models, including LASSO, RF, SVM-RFE, and XGBoost. A comprehensive evaluation of ROC curves across the algorithms demonstrated that the PLS model achieved the highest AUC among the evaluated models (AUC = 0.838), followed by the RF model (AUC = 0.760) and the discriminant model (AUC = 0.739) (Figure 4B). These results suggested that the selected candidate genes had potential discriminative value for distinguishing CD from control samples at the bulk transcriptomic level.

To further interpret the contribution of individual genes to model prediction, SHAP analysis was performed. The results indicated that *CALD1* and *CALM1* were the two features with the highest contribution to the model’s predictive output (Figure 4C–G). Their expression patterns were then examined in both the GSE186582 training cohort and the GSE66407 independent external validation cohort. The results revealed that, compared with the healthy control group, *CALD1* was upregulated in CD intestinal tissues, whereas *CALM1* was significantly downregulated (*p* < 0.05) (Figure 4H,I). This consistent differential expression across distinct cohorts suggests that *CALD1* and *CALM1* may have potential value as core lactylation-score-associated transcriptional candidate biomarkers in CD.

### 2.4. Identification and Functional Annotation of CALD1 and CALM1 Co-Expression Network Modules

**Figure 5:**
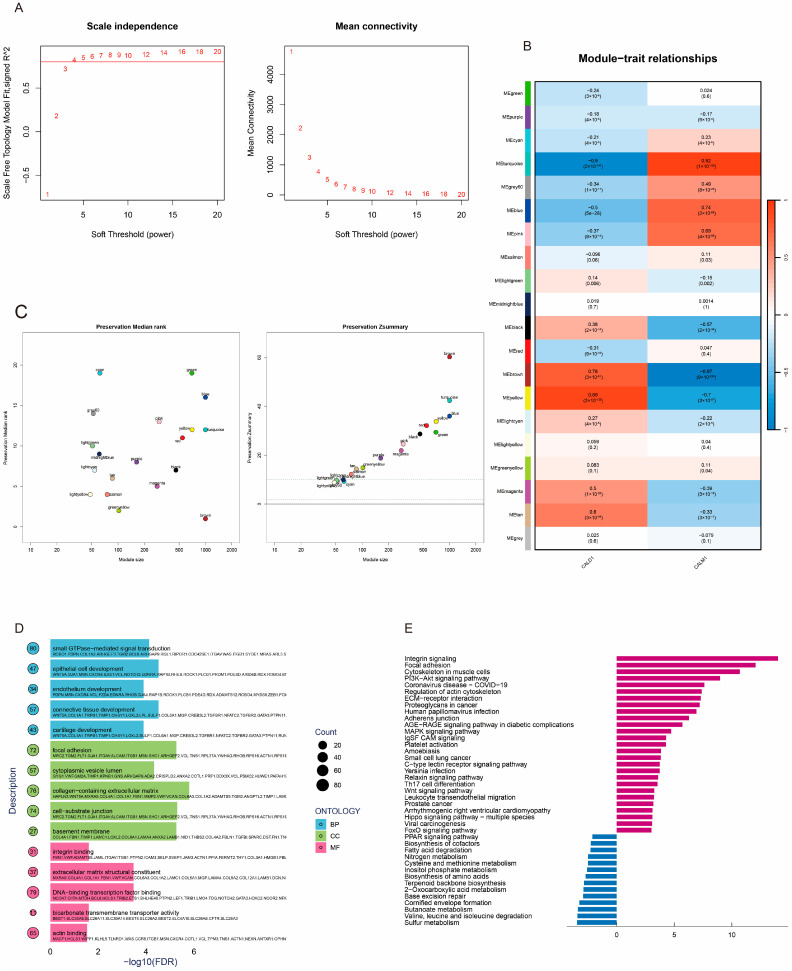
Identification and functional enrichment analysis of *CALD1* and *CALM1* co-expression modules. (**A**) Selection of the soft-thresholding power for WGCNA network construction. The red horizontal line indicates the predefined scale-free topology fit threshold of *R*^2^ = 0.8. (**B**) Heatmap of module–trait relationships (illustrating the correlations between the identified co-expression modules and the expression levels of *CALD1* and *CALM1*). (**C**) Module preservation analysis (the left panel displays the median rank, and the right panel presents the Zsummary statistic). In the right panel, the gray dotted line indicates the *Z*summary = 10 threshold for strong module preservation, whereas the gray solid line denotes the *Z*summary = 0 reference line. (**D**) GO enrichment analysis of genes within the critical co-expression modules. (**E**) Pathway enrichment analysis of genes within the critical co-expression modules. Magenta bars indicate pathways with positive enrichment scores, whereas blue bars indicate pathways with negative enrichment scores; bar length represents the magnitude of the enrichment score. In panel (**A**), β = 4 was selected for bulk-WGCNA based on the scale-free topology model fit index (R^2^ = 0.823).

To elucidate the potential molecular regulatory networks of *CALD1* and *CALM1*, a weighted gene co-expression network was constructed based on the WGCNA algorithm. To satisfy the criteria of a scale-free topological network, an optimal soft-thresholding power of four was selected for network construction (Figure 5A). At β = 4, the scale-free topology model fit index was R^2^ = 0.823, meeting the predefined scale-free topology fit criterion of 0.8. The expression matrix was subsequently partitioned into multiple gene modules via dynamic tree cutting.

Module–trait relationship analysis demonstrated that the expression level of *CALD1* was significantly positively correlated with the yellow module (MEyellow) and the brown module (MEbrown), whereas *CALM1* exhibited a robust negative correlation with these two modules (Figure 5B). Furthermore, module preservation analysis confirmed that these critical modules possessed excellent stability within the validation dataset (Zsummary > 10) (Figure 5C). These results suggested that MEyellow and MEbrown may represent *CALD1/CALM1*-associated co-expression modules suitable for subsequent functional annotation.

To delineate the biological functions of these core modules, Gene Ontology (GO) and Kyoto Encyclopedia of Genes and Genomes (KEGG) enrichment analyses were subsequently conducted. To account for multiple testing, *p*-values from GO and KEGG enrichment analyses were adjusted using the Benjamini–Hochberg false discovery rate method. Only GO terms and KEGG pathways with an adjusted *p* value/FDR < 0.05 were considered statistically significant. GO annotation revealed that the intramodular genes were predominantly enriched in biological processes such as epithelial cell development, focal adhesion, and integrin binding (Figure 5D). Pathway enrichment analysis further disclosed that these genes were significantly involved in the integrin signaling pathway, focal adhesion, and the PI3K-Akt signaling pathway (Figure 5E). These findings suggest that *CALD1* and *CALM1* may be linked to epithelial adhesion, cytoskeletal organization, and signaling pathways relevant to epithelial remodeling in CD.

### 2.5. Construction and Evaluation of a Diagnostic Nomogram Based on CALD1 and CALM1

**Figure 6:**
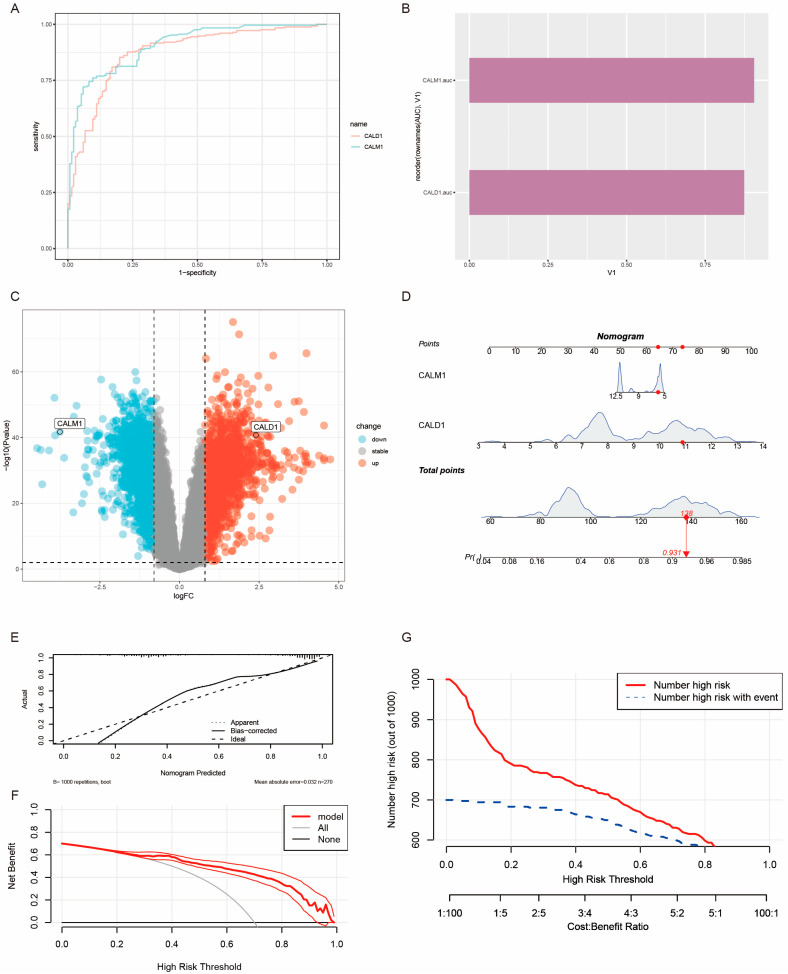
Evaluation of diagnostic efficacy and the construction and validation of a clinical predictive nomogram based on *CALD1* and *CALM1*. (**A**) ROC curve analysis of the core genes. (**B**) Comparison of the AUC values between *CALD1* and *CALM1*. (**C**) Volcano plot illustrating the differential expression profiles of the core genes. The vertical dashed lines indicate the log2 fold-change cutoffs of −1 and 1, and the horizontal dashed line indicates the adjusted *p*-value threshold of 0.05. (**D**) Diagnostic nomogram constructed based on the expression levels of *CALD1* and *CALM1*. The red dots indicate an illustrative set of predictor values and their corresponding points, and the red arrow maps the total points to the predicted probability of CD status. (**E**) Calibration curve of the nomogram model. (**F**) DCA of the nomogram model. (**G**) CIC of the nomogram model. The thick red curve represents the estimated net benefit of the CALD1/CALM1-based nomogram, whereas the thinner red curves indicate the corresponding uncertainty limits. The gray and black lines represent the treat-all and treat-none strategies, respectively. Panel (**C**): limma with BH correction; ROC curves were used for AUC calculation.

To evaluate whether *CALD1* and *CALM1* could jointly contribute to the discrimination of CD and control samples at the bulk transcriptomic level, we constructed a diagnostic model based on their expression profiles. Prior to the construction of the diagnostic model, quality control and batch effect removal were performed on the merged dataset to correct systematic errors among samples, thereby ensuring the consistency of expression distributions and the reliability of downstream analyses (Supplementary Figure S2A). Differential expression analysis corroborated that, compared with the healthy control group, *CALD1* was significantly upregulated in the intestinal tissues of CD patients, whereas *CALM1* was remarkably downregulated (Figure 6C).

We first assessed the individual diagnostic performance of *CALD1* and *CALM1* using ROC curve analysis. The results demonstrated that both genes possessed favorable diagnostic efficacy, and the AUC of *CALM1* was higher than that of *CALD1* (Figure 6A,B). Based on the expression levels of these two genes, a clinical diagnostic nomogram model was constructed to estimate the predicted probability of CD status (Figure 6D). By integrating the expression profiles of *CALD1* and *CALM1*, this model calculates a total risk score to intuitively quantify an individual’s predicted probability of CD status. Model validation results revealed that the predicted risk in the calibration curve was highly consistent with the actual observed probability, closely aligning with the ideal diagonal line (Figure 6E), which suggests that the model possesses acceptable calibration. To further assess potential overfitting, internal validation was performed using 1000 bootstrap resampling iterations. The apparent C-index/AUC was 0.893, and the optimism-corrected C-index/AUC was 0.892. The apparent and optimism-corrected Brier scores were 0.132 and 0.135, respectively, supporting acceptable discrimination and calibration performance of the nomogram. DCA demonstrated that across a wide range of threshold probabilities, the clinical net benefit based on this nomogram was superior to the extreme strategies of “intervening in all patients” (treat-all) or “intervening in none” (treat-none) (Figure 6F). Concurrently, the CIC showed that the number of high-risk individuals classified by the model was generally consistent with the actual number of confirmed cases (Figure 6G). These results suggest that the *CALD1*/*CALM1*-based nomogram has potential diagnostic value for distinguishing CD from control samples in transcriptomic cohorts.

### 2.6. Validation of CALD1 and CALM1 in Clinical Intestinal Tissues and a TNBS-Induced Murine Colitis Model

**Figure 7:**
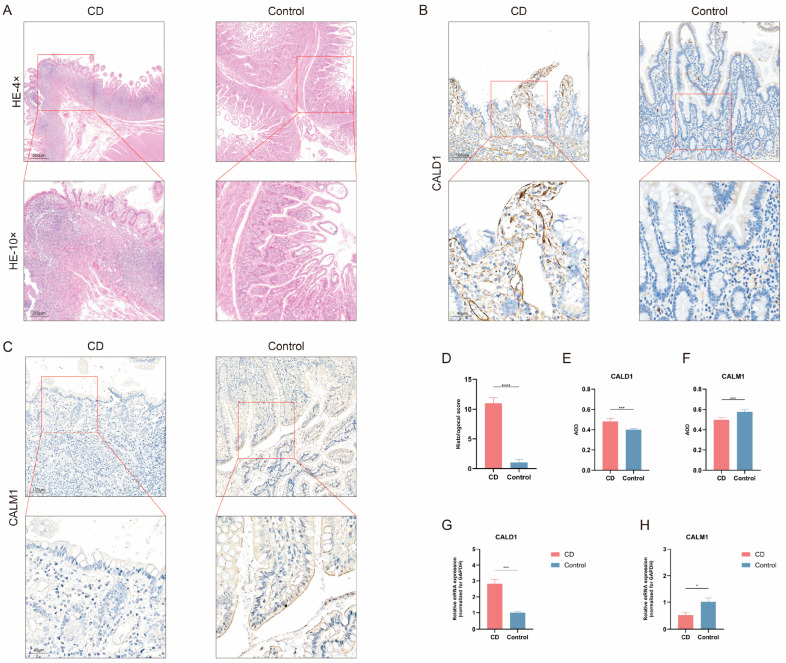
Validation of *CALD1* and *CALM1* expression in intestinal tissues of patients with Crohn’s disease. (**A**) Representative H&E staining images of intestinal tissues from healthy control (Ctrl) and CD patients. Scale bars represent 500 μm for 4× images and 200 μm for 10× images. (**B**) IHC staining images of *CALD1* in intestinal tissues from Ctrl and CD patients. (**C**) IHC staining images of *CALM1* in intestinal tissues from Ctrl and CD patients. (**D**) Quantitative comparison of histological scores in the intestinal tissues. In panels B and C, the scale bars represent 100 μm in the low-magnification images and 40 μm in the high-magnification images. (**E**) Quantitative comparison of the AOD values for *CALD1*-positive staining in the intestinal tissues. (**F**) Quantitative comparison of the AOD values for *CALM1*-positive staining in the intestinal tissues. (**G**,**H**) qRT-PCR validation of *CALD1* and *CALM1* mRNA expression in human intestinal tissues. Panels (**D**–**H**): unpaired Student’s *t*-test or Mann–Whitney U test. * *p* < 0.05; *** *p* < 0.001; **** *p* < 0.0001.

After evaluating the diagnostic relevance of *CALD1* and *CALM1* in transcriptomic cohort, we next examined whether their expression changes could be observed in clinical intestinal tissues and an in vivo murine colitis model. In human intestinal tissues, H&E staining revealed that intestinal tissues from CD patients exhibited typical pathological changes, including inflammatory cell infiltration, crypt architectural distortion, and mucosal injury, accompanied by significantly increased histological scores compared with controls (Figure 7A,D). qRT-PCR analysis showed that *CALD1* mRNA expression was significantly increased in CD tissues, whereas *CALM1* mRNA expression was decreased (Figure 7G,H). Furthermore, to determine whether these transcriptional differences were reflected at the protein level, IHC staining combined with AOD analysis was performed. Low-level basal *CALD1* staining could be detected in parts of the control mucosa; however, CD tissues showed stronger and more extensive *CALD1*-positive DAB staining, particularly in lamina propria-associated and mucosal remodeling areas (Figure 7B). This observation was supported by increased AOD values for CALD1 staining in CD tissues (Figure 7E). In contrast, *CALM1*-positive DAB staining was mainly observed in mucosal epithelial regions. Compared with control tissues, CD tissues showed weaker *CALM1* immunoreactivity (Figure 7C), which was further supported by AOD quantification (Figure 7F).

**Figure 8:**
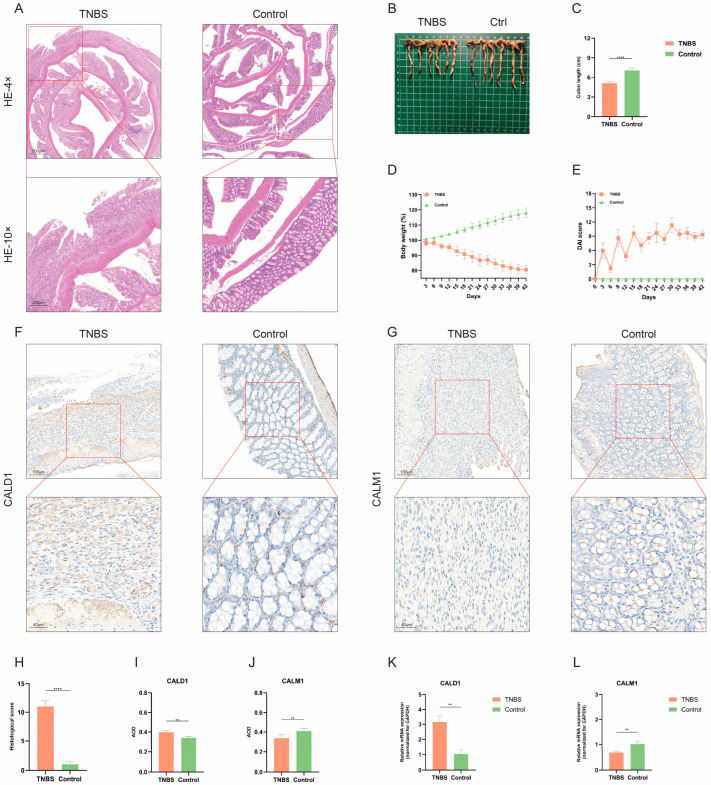
Validation of *CALD1* and *CALM1* expression in a TNBS-induced murine colitis model. (**A**) Representative H&E staining images of colon tissues from the control and TNBS-induced colitis mice. Scale bars represent 500 μm for 4× images and 200 μm for 10× images. (**B**) Representative macroscopic images of colons from each group. (**C**) Quantitative comparison of colon lengths across the groups. (**D**) Body weight change curves of the mice. (**E**) DAI score curves over the 21-day period. (**F**) IHC staining images of *CALD1* in colon tissues from the Ctrl and TNBS groups. (**G**) IHC staining images of *CALM1* in colon tissues from the Ctrl and TNBS groups. In panels F and G, the scale bars represent 100 μm in the low-magnification images and 40 μm in the high-magnification images. (**H**) Quantitative comparison of histological scores of the colon tissues. (**I**) Quantitative comparison of the AOD values for *CALD1*-positive staining in the colon tissues. (**J**) Quantitative comparison of the AOD values for *CALM1*-positive staining in the colon tissues. (**K**,**L**) qRT-PCR validation of *CALD1* and *CALM1* mRNA expression in mouse colon tissues. IHC quantification was based on the average of multiple sections and random non-overlapping high-power fields per animal two-group comparisons: unpaired Student’s *t*-test or Mann–Whitney U test; body weight and DAI: two-way repeated-measures ANOVA with Sidak’s test. ** *p* < 0.01; **** *p* < 0.0001.

To further examine whether similar expression changes could be observed in an in vivo inflammatory colitis model, we performed parallel validation in a TNBS-induced murine colitis model. Compared with control mice, TNBS-induced mice exhibited shortened colon length, progressive body-weight loss, increased disease activity index scores, severe epithelial injury, inflammatory infiltration, crypt architectural disruption, mucosal edema, and significantly elevated histological scores, confirming successful model establishment (Figure 8A–H). IHC staining showed *CALD1*-positive DAB staining was stronger in TNBS-treated colon tissues than in control tissues, whereas *CALM1*-positive staining was relatively reduced in the TNBS group (Figure 8F,G). Consistently, AOD-based quantitative analysis showed increased *CALD1* expression and decreased *CALM1* expression in TNBS-treated mice (Figure 8I,J). qRT-PCR analysis further confirmed increased *cald1* mRNA expression and decreased *calm1* mRNA expression in mouse colon tissues from the TNBS group (Figure 8K,L).

These clinical and animal-model validation results were consistent with the transcriptomic cohort findings, supporting CALD1 upregulation and CALM1 downregulation as reproducible expression patterns associated with CD intestinal inflammation and tissue remodeling.

### 2.7. Association of CALM1 Expression with Anti-TNF-α Treatment Response

**Figure 9:**
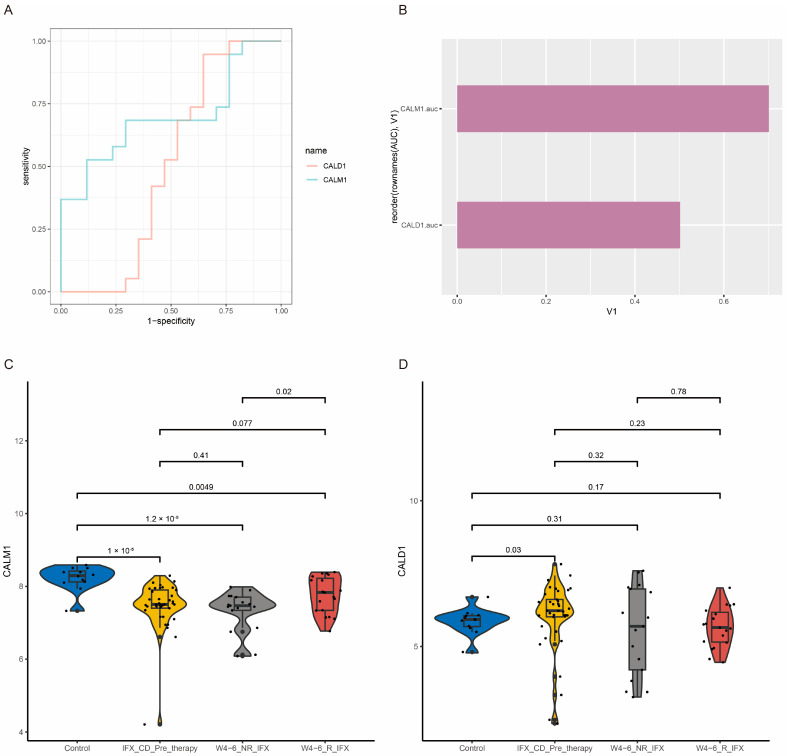
Evaluation of the predictive efficacy of *CALD1* and *CALM1* for anti-TNF-α treatment response in Crohn’s disease patients. (**A**) ROC curves of the core genes for predicting the response to IFX treatment. (**B**) Comparison of the AUC values between *CALD1* and *CALM1*. (**C**,**D**) Violin plots illustrating the expression distributions of *CALM1* (**C**) and *CALD1* (**D**) across populations with distinct clinical statuses: healthy controls (control), CD patients prior to IFX treatment (IFX_CD_Pre_therapy), non-responders at 4–6 weeks post-treatment (W4-6_NR_IFX), and responders at 4–6 weeks post-treatment (W4-6_R_IFX). Panels (**C**,**D**): Kruskal–Wallis test with Dunn’s post hoc test; panels (**A**,**B**): ROC-based AUC calculation.

Building upon the diagnostic relevance of *CALD1* and *CALM1* in CD, this study further investigated the association between these genes and the therapeutic response to the biologic agent infliximab (IFX). Through retrospective analysis of transcriptomic data from CD patients receiving IFX treatment in the GSE16879 cohort, patients were stratified into responders (Rs) and non-responders (NRs) based on clinical outcomes. ROC curve analysis demonstrated that *CALM1* had a higher AUC than *CALD1* for distinguishing IFX treatment response status (Figure 9A,B), suggesting that *CALM1* expression may be associated with favorable anti-TNF-α treatment response, whereas the specificity of *CALD1* in predicting drug response was relatively limited.

We next compared the expression patterns of *CALM1* and *CALD1* across control samples, pre-treatment CD samples, IFX non-responders, and IFX responders. Compared with the healthy control group, *CALM1* expression was downregulated in CD patients prior to treatment (pre-therapy). Following 4–6 weeks of IFX therapy, *CALM1* levels in the intestinal mucosa of the responder group (W4-6_R_IFX) increased and were significantly higher than those in the non-responder group (W4-6_NR_IFX) (Figure 9C). This expression pattern suggests that increased *CALM1* expression after IFX therapy may be associated with a favorable treatment response, rather than indicating complete reversal of the CD molecular state. Conversely, although *CALD1* showed higher expression in CD patients, it did not display a clear responder-specific expression pattern after IFX treatment (Figure 9D).

These findings suggest that *CALM1* expression may be associated with anti-TNF-α treatment response, whereas *CALD1* appears less specific for distinguishing responders from non-responders in this cohort.

### 2.8. Pseudotime Trajectory and Cell–Cell Communication Analysis of Enterocytes with High Lactylation-Associated Transcriptional Scores

**Figure 10:**
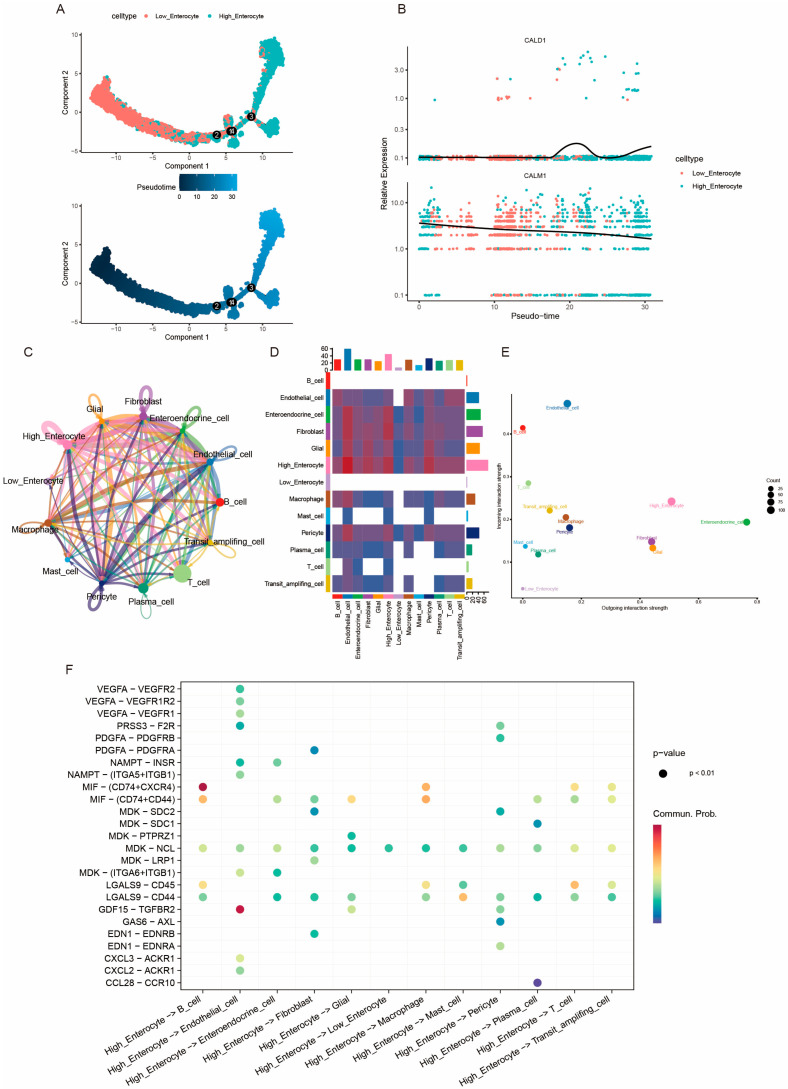
Pseudotime trajectory inference and intercellular communication network analysis of enterocytes. (**A**) Inferred pseudotime trajectory of enterocytes based on Monocle 2. The numbers indicate different trajectory branch-point identifiers identified by Monocle 2. (**B**) Expression dynamics curves of *CALD1* and *CALM1* along the pseudotime progression. (**C**) Chord diagram of the intercellular communication network. (**D**) Heatmap illustrating the number of intercellular interactions. (**E**) Scatter plot of cellular communication roles. (**F**) Dot plot of critical ligand–receptor interactions. The color gradient represents the inferred communication probability, ranging from lower values in blue to higher values in red.

To explore potential transcriptional state ordering within the enterocyte compartment, pseudotime trajectory analysis was performed using Monocle 2 (Figure 10A). The trajectory plot revealed that enterocytes were ordered from a control-enriched initial state into two distinct branches, one of which was predominantly enriched with a substantial number of High_Enterocytes with high lactylation-associated transcriptional scores derived from CD samples. As pseudotime progressed, the expression of *CALD1* exhibited an upward trend, peaking at the terminus of the trajectory, whereas the expression of *CALM1* declined early in the trajectory and remained persistently low (Figure 10B). These expression dynamics suggest that *CALD1* upregulation and *CALM1* downregulation may be associated with the transcriptional transition of enterocytes toward a CD-associated high-lactylation-score epithelial state.

We next used CellChat to infer potential cell–cell communication patterns involving High_Enterocytes. The chord diagram (Figure 10C) and the heatmap of interaction quantities (Figure 10D) demonstrated extensive communication among various cellular subpopulations within the microenvironment, with communication patterns undergoing significant alterations under disease conditions. Analysis of outgoing interaction strength revealed that High_Enterocytes possessed higher outgoing signal strength than Low_Enterocytes, suggesting their potential role as an important sender population in the CD intestinal microenvironment (Figure 10E).

Ligand–receptor interaction analysis further identified the potential molecular communication mechanisms of High_Enterocytes (Figure 10F). Among them, *GDF15–TGFBR2* interaction was mainly linked to endothelial cells, suggesting a potential epithelial–endothelial communication route associated with vascular remodeling. In addition, the *MIF–CD74/CXCR4* axis connected High_Enterocytes with macrophages and B cells, suggesting that High_Enterocytes may participate in immune-cell-associated communication, particularly macrophage-related inflammatory responses, through MIF-related signaling. Furthermore, a comparative assessment of the communication capacity of Low_Enterocytes revealed that this subpopulation exhibited relatively low communication activity in the aforementioned pathways (Supplementary Figure S3A).

These pseudotime and CellChat analyses suggest that High_Enterocytes are associated with altered *CALD1*/*CALM1* expression dynamics and may participate in epithelial–vascular–immune communication through *GDF15*- and *MIF*-related signaling.

### 2.9. Spatial Transcriptomic Mapping of High_Enterocytes and Epithelial–Vascular–Immune Neighborhoods

**Figure 11:**
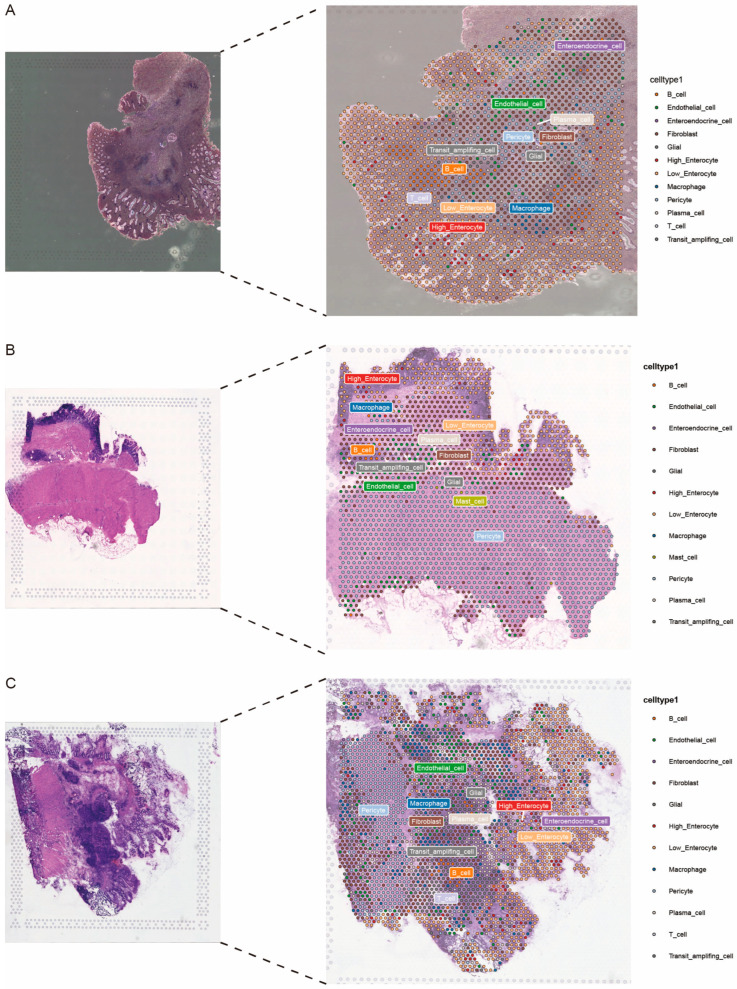
Spatial transcriptomic mapping of High_Enterocytes and epithelial–vascular–immune neighborhoods. (**A**–**C**) Spatial transcriptomics analysis of three representative intestinal tissue sections from CD patients. The left panels display the H&E staining images, while the right panels present the corresponding spatial spot-based cell-type mapping. Within the mapping, red spots represent High_Enterocytes, green spots represent Endothelial_cells, and light blue spots represent macrophages.

To further examine the spatial distribution of the High_Enterocytes state within CD intestinal tissue, we leveraged spatial transcriptomics to perform in situ analysis on CD intestinal tissue sections. By mapping scRNA-seq-defined cell populations onto tissue sections, we constructed a spatial distribution landscape (Figure 11A–C). Spatial transcriptomic mapping showed that High_Enterocytes were mainly enriched in diseased mucosal epithelial regions of CD tissue sections, particularly in areas with epithelial disruption and inflammatory remodeling. Compared with Low_Enterocytes, High_Enterocytes displayed a more localized enrichment pattern within lesion-associated epithelial regions and were frequently observed near macrophage-, endothelial-cell-, and fibroblast-enriched areas. In contrast, Low_Enterocytes were more broadly distributed across enterocyte-associated epithelial regions.

We further examined the spatial relationship between High_Enterocytes-enriched regions and other microenvironmental cell populations. As illustrated in Figure 11A–C, the edges or interior of zones enriched with High_Enterocytes were accompanied by endothelial cells and macrophages. This spatial pattern is consistent with the CellChat-based inference that High_Enterocytes may participate in epithelial–vascular–immune communication involving GDF15- and MIF-related signaling. Because the spatial transcriptomic data were analyzed at spot-level resolution, these results should be interpreted as spatial enrichment patterns of cell-state signatures rather than exact single-cell localization.

Together, these findings provide spatial-level support for a localized epithelial–vascular–immune neighborhood involving High_Enterocytes and macrophage-, endothelial-cell-, and fibroblast-enriched regions in CD lesions.

## 3. Discussion

The pathogenesis of CD represents a complex immune cascade triggered by the interplay of genetic susceptibility, environmental cues, and dysbiosis [23,24]. While the role of exogenous microbial metabolites in immune regulation has been extensively documented, the impact of endogenous metabolites, specifically lactate-associated transcriptional and epigenetic remodeling, on the intestinal microenvironment remains in its infancy [25,26]. To address intestinal fibrosis, a core complication driving high surgical rates in CD, and the clinical dilemma of “inflammation–fibrosis decoupling” [27], this study explored lactylation-associated transcriptional remodeling within the fibrotic network, through integrated multi-omics analysis. Our findings offer a potential metabolism–epigenetics–microenvironment crosstalk perspective for understanding why established fibrosis may persist despite effective inflammatory control. Ultimately, this work lays a solid theoretical foundation for the development of novel targets for the prediction and therapeutic intervention of CD-associated fibrosis.

Previous studies on CD have predominantly focused on fibroblast activation or immune-cell infiltration. In this study, we focused on the enterocyte compartment because scRNA-seq analysis revealed a High_Enterocytes state with relatively elevated lactylation-associated transcriptional scores and marked transcriptional heterogeneity in CD lesions. Within this epithelial state, the five genes shown in Figure 3C, including CALM1, CALD1, PTMA, ALDOB, and PPIA, were initially defined as lactylation-related candidate feature genes because they were derived from the intersection between a predefined lactate metabolism-/lactylation-related gene set and High_Enterocytes-associated hdWGCNA module genes. This definition reflects their transcriptional- and signature-level association with lactate/lactylation-related programs, rather than direct evidence that all five encoded proteins undergo site-specific lysine lactylation in CD intestinal tissues. Among them, *ALDOB* has been reported to undergo site-specific lysine lactylation and participate in metabolic reprogramming [28], whereas *PPIA*, *PTMA*, *CALD1*, and *CALM1* have mainly been reported in lactylation-related proteomic datasets, disease signatures, or hub-gene analyses, supporting their association with lactylation-related phenotypes [28,29,30]. By integrating hdWGCNA and machine learning algorithms, we identified *CALD1* and *CALM1* as core lactylation-score-associated transcriptional biomarkers of this epithelial state. *CALD1* encodes an actin cytoskeleton regulator, and its upregulation was associated with epithelial structural remodeling [31]. Conversely, *CALM1* encodes calmodulin, acting as a calcium sensor to maintain calcium homeostasis and downstream signaling; its downregulation is closely linked to uncontrolled inflammation and impaired intestinal barrier function [30,32]. These findings suggest that epithelial transcriptional remodeling may be an important component of the CD intestinal microenvironment, while direct protein lactylation or histone lactylation events remain to be experimentally validated.

Mechanistically, *CALD1*/*CALM1* dysregulation was associated with altered paracrine signaling from High_Enterocytes to neighboring vascular and immune cells. Cell communication analysis suggested two major epithelial-centered axes: *GDF15*–*TGFBR2*, potentially linked to endothelial remodeling [33,34], and *MIF*–*CD74*/*CXCR4*, potentially linked to macrophage recruitment or activation [35,36]. Importantly, macrophages were not treated as the primary discovery population in this study, but as important downstream immune effector cells within the High_Enterocytes-associated microenvironment. Spatial transcriptomics visually confirmed the physical co-localization of high-lactylation enterocytes, endothelial cells, and macrophages within the CD lesional areas, supporting the existence of epithelial–vascular–immune neighborhoods. However, macrophage subclusters, macrophage polarization, and macrophage-specific lactylation-associated transcriptional programs require further dedicated analysis.

In terms of clinical translation, the diagnostic nomogram constructed based on *CALD1* and *CALM1* exhibited robust evaluation efficacy in stratifying the risk of CD. Retrospective analysis of the GSE16879 cohort indicated that CALM1 expression was associated with anti-TNF-α/IFX treatment response. CALM1 expression tended to increase in IFX responders compared with non-responders, whereas CALD1 did not show a clear responder-specific expression pattern after IFX treatment. TNF-α-related inflammation may indirectly affect lactylation-associated transcriptional remodeling through tissue hypoxia, glycolytic activation, and lactate accumulation. However, because the GSE16879 cohort lacks direct measurements of lactate abundance and global or site-specific lactylation levels, the increased CALM1 expression observed in IFX responders should be interpreted as a treatment-response-associated transcriptional change rather than direct evidence of lactylation reversal.

Although this study preliminarily delineated the lactylation-associated transcriptional features related to CD intestinal fibrosis through multi-omics integration and experimental validation, several limitations remain to be addressed in future research. First, the lactylation-associated transcriptional score used in this study was inferred from transcriptomic data and should not be interpreted as a direct measurement of global or site-specific protein lactylation [14,37]. Future studies using lactylation proteomics, anti-Kla immunoprecipitation, site-specific lactylation assays, and histone lactylation ChIP-seq or CUT&Tag are required to clarify whether *CALD1*/*CALM1* are directly or indirectly regulated by lactylation [38,39]. Second, at the disease model level, although a chronic TNBS-induced chemical colitis model was employed, this model still falls short of fully recapitulating the complex, spontaneous pathological features of human Crohn’s disease [40]. Therefore, it is imperative for future research to incorporate patient-derived organoids (PDOs), gut-on-a-chip models and macrophage-specific functional assays, to reconstruct the epithelial–vascular–immune spatial niche in vitro with high fidelity, thereby further investigating validate the GDF15 and MIF communication axes [41,42]. Third, because of ethical and clinical sampling limitations, the non-IBD control tissues used for clinical validation were obtained from grossly normal, tumor-free surgical margin tissues of patients with right-sided colon adenocarcinoma rather than from completely healthy donors. Although these tissues were pathologically confirmed to be free of tumor infiltration, obvious dysplasia, and active inflammation, potential background differences associated with cancer-patient-derived normal tissues cannot be fully excluded. Finally, the predictive efficacy of *CALM1* for anti-TNF-α treatment response remains limited to retrospective cohort analyses. There is an urgent need for multi-center, large-sample prospective clinical studies to systematically evaluate the robustness of the diagnostic nomogram across diverse populations and disease phenotypes [43], and to explore whether the expression dynamics of *CALM1* are applicable for predicting the efficacy of other biologics, such as vedolizumab or ustekinumab, thereby providing a more comprehensive evidence-based foundation for the precise subtyping and individualized intervention of CD [44].

## 4. Materials and Methods

### 4.1. Study Design, Data Acquisition and Cohort Roles

**Figure 12:**
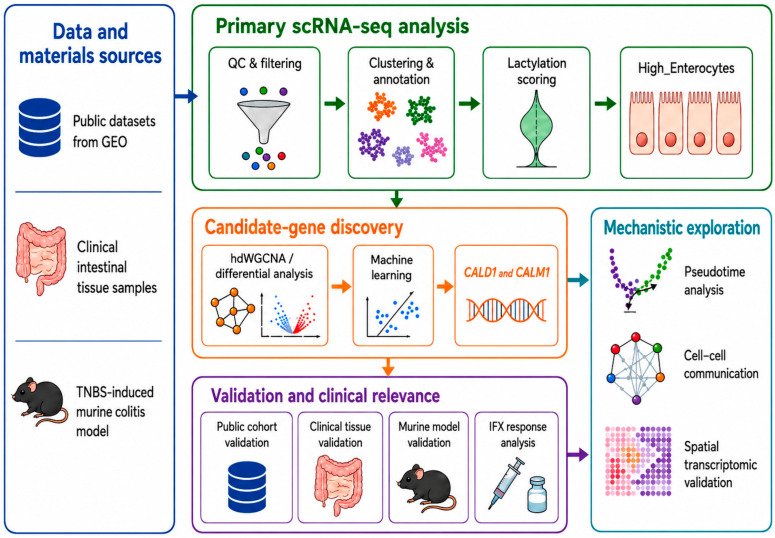
The workflow illustrates the overall design of this study. Public datasets were collected and used for single-cell transcriptomic analysis, machine learning-based biomarker screening, treatment-response evaluation, and spatial transcriptomic validation. scRNA-seq data were used to characterize the cellular landscape and identify cell populations with relatively elevated lactylation activity. Candidate lactylation-associated genes were further screened by hdWGCNA and machine learning methods, and subsequently validated in public cohorts, clinical intestinal tissue samples, and a TNBS-induced murine colitis model. Additional analyses, including pseudotime trajectory analysis, cell–cell communication analysis, and spatial transcriptomics, were performed to explore the potential microenvironmental involvement of the identified cell populations and candidate genes in CD-associated intestinal fibrosis.

The overall workflow of this study is illustrated in Figure 12. This study was designed as a sequential multi-omics analysis consisting of single-cell discovery, candidate-gene identification, cohort-level validation, experimental validation, and microenvironmental interpretation. All transcriptomic datasets used in this study were obtained from the Gene Expression Omnibus (GEO) database. The accession number, platform, data type, original dataset design, samples included in this study, inclusion/exclusion criteria, and analytical role of each dataset are summarized in Supplementary Table S1.

Public datasets were grouped according to their analytical roles. GSE282122 was used as the single-cell discovery cohort to characterize the CD intestinal cellular landscape, calculate lactylation-associated transcriptional scores, identify High_Enterocytes, and perform enterocyte-specific hdWGCNA, pseudotime, and CellChat analyses. GSE186582, GSE66407, and GSE59071 were used as bulk transcriptomic cohorts for candidate-gene modeling, external validation, and diagnostic model construction. GSE16879 was used to assess the association between core-gene expression and anti-TNF-α/infliximab treatment response. GSE228360 was used for spatial transcriptomic mapping of scRNA-seq-defined cell-type and cell-state signatures in CD intestinal tissue.

For GSE282122, although the original dataset included IBD samples related to adalimumab treatment, the present study retained only 28 CD inflamed lesion samples and 12 healthy control samples. UC samples, post-treatment samples, and samples not aligned with the purpose of this study were excluded. Therefore, GSE282122 was used for scRNA-seq-based discovery of CD-associated cellular states and lactylation-associated transcriptional features, rather than for evaluating adalimumab treatment response.

For GSE186582, the original dataset included samples from inflamed ileum at surgery (M0I), ileal margin at surgery (M0M), postoperative 6-month endoscopy samples (M6), and non-IBD controls. In the present study, only 196 CD inflamed ileal mucosal samples (M0I) and 25 non-IBD control samples were included. M0M and M6 samples were excluded because this analysis focused on cross-sectional CD-versus-control biomarker discovery rather than postoperative recurrence or longitudinal disease progression. GSE186582 was therefore used as the bulk transcriptomic training cohort. The candidate features used for machine learning modeling were derived from the independent scRNA-seq-based hdWGCNA analysis of GSE282122, rather than being selected from the full GSE186582 cohort, thereby reducing the risk of data leakage.

For merged bulk transcriptomic analyses, expression matrices were processed by probe annotation, gene–symbol conversion, log2 transformation, and normalization when applicable. Shared genes were retained, and batch effects were corrected using the ComBat algorithm from the R package sva, with dataset/platform source defined as the batch variable and disease status retained as the biological covariate. Clinical intestinal tissues and the TNBS-induced murine colitis model were used for experimental validation of core-gene expression.

### 4.2. Single-Cell RNA-Seq Preprocessing, Integration, Clustering, and Cell-Type Annotation

Single-cell RNA-seq data from GSE282122 were analyzed using R version 4.4.3 with Seurat v5.5.0, SeuratObject v5.4.0, Harmony v2.0.3, and DoubletFinder v2.0.6. After quality control and doublet removal, 312,049 high-quality cells with 24,161 detected genes/features were retained for downstream analysis. The RNA assay was used as the default assay.

Gene expression matrices were normalized using the LogNormalize method with a scale factor of 10,000. The top 2000 highly variable genes were identified using the vst method and were used for principal component analysis. Data scaling was performed using ScaleData() with centering and scaling enabled; no additional regression was performed during ScaleData(), because no variables were specified in vars.to.regress.

To reduce sample-level variation, Harmony integration was performed after PCA. The first 15 Harmony dimensions were used for downstream nearest-neighbor graph construction and UMAP visualization. Specifically, FindNeighbors() was performed using reduction = “harmony”, dims = 1:15, and k.param = 20. UMAP visualization was performed using the uwot method based on the same Harmony dimensions. Multiple clustering resolutions, including 0.01, 0.05, 0.1, 0.2, 0.3, 0.5, 0.8, and 1.0, were evaluated. A resolution of 0.1 was selected for the main analysis because it provided stable separation of major intestinal cell populations without excessive over-clustering. This resolution generated 13 clusters.

The resulting clusters were annotated into 12 major cell types according to canonical marker genes and cluster-specific marker expression patterns, including T cells, plasma cells, enterocytes, B cells, fibroblasts, macrophages, endothelial cells, glial cells, pericytes, transit-amplifying cells, enteroendocrine cells, and mast cells. Marker genes for each annotated cell type were identified using the Seurat FindAllMarkers() function with the Wilcoxon rank-sum test. Only positively enriched markers were retained by setting only.pos = TRUE. The thresholds for marker detection were min.pct = 0.25, logfc.threshold = 0.25, and adjusted *p*-value < 0.05. Cell type annotation was based on both statistically significant marker genes and canonical lineage markers to ensure biological consistency.

### 4.3. Calculation of the Lactylation-Associated Transcriptional Score

To quantify the lactylation-associated transcriptional activity at the single-cell resolution, a curated lactate metabolism- and lactylation-related gene set was used to calculate a lactylation-associated transcriptional score for each cell. The gene set included genes involved in lactate production, lactate transport, lactate utilization, candidate lactylation writer/eraser-related regulation, and lactylation-associated biological processes (Supplementary Table S2).

The lactylation-associated transcriptional score was calculated using the AddModuleScore function in Seurat based on normalized single-cell gene-expression data. Briefly, for each cell, AddModuleScore calculated the average expression of genes in the predefined lactate metabolism- and lactylation-related signature and subtracted the average expression of matched background control genes with similar expression levels. The resulting score represented the relative enrichment of the lactylation-related transcriptional signature in each individual cell. Because the input data were single-cell transcriptomic profiles, this score should be interpreted as a functional transcriptional score associated with lactate metabolism- and lactylation-related biological processes, rather than as a direct measurement of global or site-specific protein lactylation.

Cells were then compared across major cell types and disease groups according to their lactylation-associated transcriptional scores. Within the enterocyte compartment, cells with relatively higher scores were defined as High_Enterocytes for downstream analyses, whereas the remaining enterocytes were defined as Low_Enterocytes.

### 4.4. High-Dimensional Weighted Gene Co-Expression Network Analysis (hdWGCNA)

To explore gene co-expression networks associated with the High_Enterocytes state, we employed the hdWGCNA package to construct a high-dimensional gene co-expression network for the identified enterocyte subpopulation. This analysis focused on enterocytes because they showed relatively high lactylation-associated transcriptional scores and evident transcriptional heterogeneity under CD conditions. The soft-thresholding power was selected by jointly considering the scale-free topology model fit index and network connectivity. The selected soft-thresholding power was then used to construct the topological overlap matrix (TOM), and gene modules were identified using the dynamic tree cut algorithm. Module eigengenes (MEs) were calculated to summarize the expression pattern of each module and were correlated with the High_Enterocytes state, lactylation-associated transcriptional score, and CD status. Hub genes within key modules were ranked according to intra-modular connectivity (kME) values. These hub genes were subsequently used for lactylation-related candidate-gene screening and machine learning analysis.

### 4.5. Machine Learning Analysis and SHAP-Based Feature Interpretation

Candidate features for machine learning analysis were generated by intersecting the top hub genes from the High_Enterocytes-associated blue module with the predefined lactate metabolism- and lactylation-related gene set. These features were derived from the independent scRNA-seq-based hdWGCNA analysis of GSE282122 and were then evaluated in bulk transcriptomic cohorts.

GSE186582 was used as the training cohort. The input matrix consisted of candidate-gene expression values, with rows representing samples and columns representing genes. The binary outcome variable was disease status, defined as CD or control. Ten machine learning algorithms were incorporated, including Least Absolute Shrinkage and Selection Operator (LASSO) regression, Random Forest (RF), Support Vector Machine–Recursive Feature Elimination (SVM-RFE), eXtreme Gradient Boosting (XGBoost), Gradient Boosting Machine (GBM), Elastic Net, Ridge Regression, Neural Network, Logistic Regression, and k-Nearest Neighbors (KNNs). Hyperparameter tuning and model comparison were performed within the GSE186582 training cohort using cross-validation; nested cross-validation was not applied. Subsequently, the predictive performance of the established models was comprehensively evaluated in an independent testing cohort (GSE66407) by calculating metrics such as the Area Under the Receiver Operating Characteristic (ROC) curve (AUC). The primary model outputs included predicted disease probabilities, classification labels, ROC-/AUC-based performance metrics, and feature-importance rankings.

SHAP analysis was performed to interpret the contribution of individual genes to model prediction. Candidate genes with high SHAP importance and consistent expression patterns across cohorts were selected as core candidate biomarkers. Because candidate features were derived from GSE282122 rather than selected from the full GSE186582 training cohort, this workflow reduced the risk of data leakage.

### 4.6. Weighted Gene Co-Expression Network Analysis (WGCNA) and Functional Enrichment

Bulk transcriptome-based WGCNA was performed to identify co-expression modules associated with *CALD1* and *CALM1*. Unlike the enterocyte-specific hdWGCNA analysis, this analysis was conducted using bulk transcriptomic expression profiles to characterize the broader transcriptomic context of the two core candidate genes. Module eigengenes were correlated with *CALD1* and *CALM1* expression levels to identify core gene-associated modules, and module preservation analysis was used to evaluate their stability in the validation dataset.

Genes within the selected modules were subjected to GO and KEGG enrichment analyses. *p* values were adjusted using the Benjamini–Hochberg false discovery rate method, and terms or pathways with adjusted *p* value/FDR < 0.05 were considered statistically significant. These enrichment analyses were used for functional annotation of the associated co-expression modules and were not intended to identify lactylation-specific pathways or direct protein lactylation events.

### 4.7. Construction and Evaluation of the Diagnostic Nomogram

To evaluate the combined diagnostic value of *CALD1* and *CALM1*, a logistic regression-based diagnostic nomogram was constructed using their expression levels as predictors and CD or control status as the binary outcome variable. The nomogram was designed to estimate the predicted probability of CD status based on the combined expression pattern of the two core candidate genes.

Model discrimination was assessed using ROC curve analysis and the AUC. Model calibration was evaluated using a calibration curve to compare predicted probabilities with observed outcomes. To assess potential optimism caused by internal validation, bootstrap resampling with 1000 repetitions was performed. The optimism-corrected C-index/AUC and Brier score were calculated to evaluate discrimination and calibration performance after correction for overfitting.

Decision curve analysis (DCA) was used to estimate the potential net benefit of the nomogram across different threshold probabilities, and clinical impact curve analysis was performed to compare the number of individuals classified as high probability with the number of observed CD cases. These analyses were used to assess the diagnostic performance of the nomogram at the transcriptomic cohort level.

### 4.8. Clinical Tissue Sample Collection

This study included surgically resected intestinal specimens from CD patients and non-IBD surgical control tissues. Written informed consent was obtained from all participating patients prior to inclusion. The study protocol was reviewed and approved by the Medical Ethics Committee of the Second Xiangya Hospital of Central South University (Approval No.: 2017-S117).

The diagnosis of CD was established based on a combination of endoscopic manifestations, histopathological findings, clinical symptoms, laboratory tests, and radiological features. CD intestinal specimens were obtained from surgically resected intestinal tissues of patients with confirmed CD. Control intestinal tissues were obtained from grossly normal, non-tumorous intestinal regions of patients undergoing radical surgery for right-sided colon adenocarcinoma. The control tissues were collected from tumor-free resection margins away from the tumor lesion, rather than from tumor tissues or immediately adjacent tumor tissues. All control tissues were confirmed by postoperative pathological examination to be free of tumor infiltration, obvious dysplasia, and active inflammation. None of the control patients received preoperative anti-tumor treatment, including neoadjuvant chemotherapy, radiotherapy, immunotherapy, or targeted therapy.

### 4.9. Animal Experiments

All experimental mice in this study were housed in a specific pathogen-free (SPF) barrier facility. All animal experimental protocols were reviewed and approved by the Experimental Animal Ethics Committee of Central South University (approval no.: CSU-2023-0110).

A 2,4,6-trinitrobenzenesulfonic acid (TNBS, Meilunbio, Dalian, China)-induced murine colitis model was established to validate the expression patterns of the core candidate genes in vivo. Eight-week-old female SPF C57BL/6 mice were randomly assigned to TNBS model group or the control group (*n* = 6 per group). For model induction, a six-cycle, dose-escalating intrarectal TNBS administration protocol was used. TNBS was dissolved in 45% ethanol and administered at 1.0% on days 0 and 7, 1.5% on days 14 and 21, and 2.0% on days 28 and 35 [45,46,47]. Mice in the control group received the corresponding vehicle treatment. Body weight, stool consistency, and fecal blood were monitored during the modeling period. At the end of the experiment, mice were sacrificed, and colon tissues were collected for colon-length measurement, histological examination, immunohistochemical staining, and qRT-PCR analysis.

Disease activity index (DAI) was used to evaluate the severity of colitis during model establishment. DAI was evaluated based on three parameters: body weight loss, stool consistency, and fecal blood/hematochezia. Each parameter was scored from 0 to 4, and the final DAI score was calculated as the sum of the three parameters: DAI = body weight loss score + stool consistency score + fecal blood score. Thus, the total DAI score ranged from 0 to 12, with higher scores indicating more severe colitis activity. Detailed scoring criteria are provided in Supplementary Table S3.

### 4.10. Quantitative Real-Time Polymerase Chain Reaction (qRT-PCR)

qRT-PCR was performed to validate the mRNA expression levels of *CALD1* and *CALM1* in human intestinal tissues and the corresponding mouse genes, *cald1* and *calm1*, in colon tissues from the TNBS-induced murine colitis model.

Total RNA was extracted from tissue samples using TRIzol reagent (NCM Biotech, Suzhou, China) according to the manufacturer’s instructions. RNA concentration and purity were assessed using a NanoDrop spectrophotometer (Thermo Fisher Scientific, Waltham, MA, USA). The extracted RNA was reverse-transcribed into cDNA utilizing the Hifair III 1st Strand cDNA Synthesis SuperMix for qPCR Kit (Yeasen, Shanghai, China). Subsequently, the Hieff qPCR SYBR Green Master Mix Kit (Yeasen, Shanghai, China) was employed for the qRT-PCR validation of target gene expression, and relative mRNA expression levels were calculated using the 2−ΔΔCt method. Gene-specific primers used for qRT-PCR in human and mouse samples are detailed in Supplementary Table S4.

### 4.11. Immunohistochemistry (IHC) and AOD Quantification

IHC was performed to assess the protein-expression patterns of *CALD1* and *CALM1* in human intestinal tissues and mouse colon tissues. Tissue specimens were fixed in 4% paraformaldehyde, routinely embedded in paraffin, and sectioned at a thickness of 4 µm. Following deparaffinization and rehydration, antigen retrieval was performed using a citrate buffer. The sections were then incubated with 3% H_2_O_2_ at room temperature for 10 min to quench endogenous peroxidase activity. Primary antibodies (*CALD1* antibody: Proteintech, Wuhan, China, 20887-1-AP, 1:2000; *CALM1* antibody: Proteintech, Wuhan, China, 10541-1-AP, 1:150) were applied dropwise and incubated overnight at 4 °C. The following day, sections were incubated with an HRP-conjugated secondary antibody (goat anti-rabbit IgG, ZSGB-BIO, Beijing, China, 1:500) at room temperature for 30 min. Color development was achieved using 3,3′-diaminobenzidine (DAB), followed by counterstaining with hematoxylin. Finally, the sections were dehydrated, cleared, and mounted with neutral resin.

Representative images were acquired under identical microscope settings within each staining batch. For quantitative analysis, positive staining was assessed using ImageJ software (version 1.37) based on the average optical density (AOD). Comparable mucosal regions of interest were selected between groups, and local enlarged images were used only to display representative staining areas rather than to define the entire quantification region. For animal samples, at least three representative colon sections per animal and at least three randomly selected non-overlapping high-power fields per section were analyzed. The same threshold settings were applied to all images, and the final AOD value for each sample or animal was calculated as the average value of the analyzed fields and was used for statistical comparison.

### 4.12. Hematoxylin and Eosin (H&E) Staining and Histological Scoring

H&E staining was performed to evaluate intestinal tissue morphology in human intestinal samples and mouse colon tissues. Following deparaffinization and rehydration, tissue sections were stained with hematoxylin for 5 min, differentiated in acid alcohol for 30 s, and stained with eosin for 3 min. The sections were then dehydrated, cleared, mounted with neutral resin, and examined under a light microscope.

Histological injury was evaluated using a modified semi-quantitative scoring system adapted from previously published experimental colitis scoring criteria. The scoring system included four parameters: epithelial damage, inflammatory infiltration, crypt architecture, and mucosal edema. Each parameter was scored from 0 to 3, representing absent/normal, mild, moderate, and severe pathological changes, respectively. A comprehensive score was calculated by summing the four parameters, with a possible range of 0–12; higher scores indicated more severe intestinal tissue damage.

All sections were independently evaluated by two investigators who were blinded to group allocation. In cases of discrepant scores, the sections were jointly re-evaluated until a consensus score was reached. Detailed scoring criteria are provided in Supplementary Table S5.

### 4.13. Analysis of Anti-TNF-α Treatment Response

The GSE16879 dataset was used to assess the association between core-gene expression and IFX treatment response in CD patients. Patients were classified as responders or non-responders according to the clinical response information provided in the original dataset. The expression levels of *CALD1* and *CALM1* were compared among control samples, pre-treatment CD samples, IFX non-responders, and IFX responders. Violin plots were used to visualize expression differences among groups. ROC curve analysis and AUC calculation were performed to evaluate the ability of each gene to distinguish responders from non-responders. This analysis was interpreted as an association between gene expression and IFX response based on retrospective transcriptomic data.

### 4.14. Cell Communication and Pseudotime Trajectory Analysis

CellChat (version 1.6.1) analysis was performed to infer potential cell–cell communication patterns involving High_Enterocytes and other cell populations in the CD intestinal microenvironment. Based on the ligand–receptor interaction database implemented in CellChat, communication probabilities between High_Enterocytes and other annotated cell types were calculated. Predicted interaction strength, communication roles, and representative ligand-receptor pairs were used to evaluate potential epithelial–vascular–immune communication patterns.

Pseudotime trajectory analysis was performed using Monocle 2 (R package monocle, version 2.34.0) to order enterocytes according to their transcriptional similarity and infer potential state transitions within the enterocyte compartment. The DDRTree algorithm was used for dimensionality reduction and trajectory construction. Gene-expression changes along the pseudotime axis were analyzed, with particular attention to the expression dynamics of *CALD1* and *CALM1*.

### 4.15. Spatial Transcriptomics Analysis

Spatial transcriptomic analysis was performed using the GSE228360 dataset to examine the tissue-level distribution of scRNA-seq-defined cell types and cell states in CD intestinal sections. The Seurat package was employed for the loading and processing of spatial data. The spatial gene expression matrix, tissue image, and corresponding spot-coordinate information were imported into R. After normalization, spatial spots were retained together with their original tissue coordinates for downstream visualization. Tissue architecture was identified in conjunction with corresponding H&E-stained images.

To map scRNA-seq-defined cell populations onto spatial tissue sections, we used a signature-based spatial mapping strategy. Marker genes for major cell types and key cell states, including High_Enterocytes, endothelial cells, and macrophages, were derived from the annotated scRNA-seq dataset. Only genes shared between the scRNA-seq and spatial transcriptomic datasets were retained for mapping.

For each spatial spot, module scores of the corresponding scRNA-seq-derived signatures were calculated using Seurat and projected onto the original tissue coordinates to visualize the spatial enrichment of specific cell populations or cellular states. Spatial relationships were assessed by comparing the distribution patterns and local overlap of signature-enriched spots.

### 4.16. Statistical Analysis

All statistical analyses were conducted using R (version 4.4.3) and GraphPad Prism (version 10). Continuous variables were summarized as mean ± standard deviation or median with interquartile range, depending on data distribution. Normality and variance homogeneity were assessed when appropriate. For comparisons between two independent groups, two-tailed unpaired Student’s *t*-test, Welch’s *t*-test, or Mann–Whitney U test were used according to data distribution and variance equality. For comparisons among multiple groups, one-way ANOVA followed by Tukey’s post hoc test, or a Kruskal–Wallis test followed by Dunn’s post hoc test, was applied as appropriate.

For single-cell analyses, differences in lactylation-associated transcriptional scores between CD and control groups within each cell type were assessed using the Wilcoxon rank-sum test, with Benjamini–Hochberg correction for multiple comparisons. Marker genes were identified using the Wilcoxon rank-sum test implemented in Seurat, and an adjusted *p* value < 0.05 was considered statistically significant. Correlation analyses were performed using Pearson or Spearman correlation according to data distribution.

For functional enrichment analyses, GO and KEGG enrichment *p* values were adjusted using the Benjamini–Hochberg false discovery rate method, and terms or pathways with adjusted *p* value/FDR < 0.05 were considered significant. ROC curve analysis was used to evaluate diagnostic or treatment-response-associated performance, and the AUC was calculated. For the diagnostic nomogram, model discrimination and calibration were evaluated using ROC/AUC analysis, calibration curves, 1000 bootstrap resampling, optimism-corrected C-index/AUC, Brier score, decision curve analysis, and clinical impact curve analysis.

A two-sided *p* value or adjusted *p* value < 0.05 was considered statistically significant. Significance levels were defined as follows: ns, *p* or adjusted *p* ≥ 0.05; *, *p* or adjusted *p* < 0.05; **, *p* or adjusted *p* < 0.01; ***, *p* or adjusted *p* < 0.001; ****, *p* or adjusted *p* < 0.0001.

## 5. Conclusions

By integrating single-cell transcriptomics, spatial transcriptomics, and multi-algorithm machine learning with clinical cohorts and animal models, this study characterized lactylation-associated transcriptional remodeling within the CD intestinal microenvironment. We identified *CALD1* and *CALM1* as candidate lactylation-score-associated transcriptional biomarkers linked to epithelial dysfunction and fibrotic remodeling, and highlighted potential epithelial–vascular–immune communication patterns involving *GDF15*–*TGFBR2* and *MIF*–*CD74*/*CXCR4* axes. Furthermore, our data support the potential clinical value of *CALD1* and *CALM1* as diagnostic biomarkers and of *CALM1* as a treatment-response-associated marker for anti-TNF-α therapy. This study provides a multi-omics framework for understanding the metabolic–epigenetic features of CD-associated intestinal fibrosis and offers candidate markers for future mechanistic and translational validation.

## Data Availability

All datasets analyzed in this study are publicly available in the Gene Expression Omnibus (GEO) repository (https://www.ncbi.nlm.nih.gov/geo/, accessed on 22 June 2026). Bulk transcriptomic datasets used include GSE186582, GSE66407, GSE59071 and GSE16879. Single-cell RNA sequencing and spatial transcriptomics datasets are accessible under accession numbers GSE282122 and GSE228360, respectively.

## Supplementary Materials

The following supporting information can be downloaded at https://www.mdpi.com/article/10.3390/ijms27146343/s1.

## Abbreviations

The following abbreviations are used in this manuscript:

CD	Crohn’s disease
IBD	inflammatory bowel disease
ECM	extracellular matrix
TNF-α	tumor necrosis factor-alpha
Kla	protein lactylation
scRNA-seq	single-cell RNA sequencing
hdWGCNA	high-dimensional weighted gene co-expression network analysis
GEO	Gene Expression Omnibus
PCA	Principal component analysis
UMAP	Uniform Manifold Approximation and Projection
HVGs	highly variable genes
PCs	principal components
TOM	topological overlap matrix
MEs	module eigengenes
kME	intra-modular connectivity
LASSO	Least Absolute Shrinkage and Selection Operator
RF	Random Forest
SVM-RFE	Support Vector Machine–Recursive Feature Elimination
XGBoost	eXtreme Gradient Boosting
GBM	Gradient Boosting Machine
KNNs	k-Nearest Neighbors
ROC	Receiver Operating Characteristic
AUC	Area Under the Curve
SHAP	SHapley Additive exPlanations
GO	Gene Ontology
GSEA	Gene Set Enrichment Analysis
DCA	Decision curve analysis
CIC	Clinical impact curve
SPF	Specific pathogen-free
TNBS	2,4,6-trinitrobenzenesulfonic acid
DAI	Disease Activity Index
qRT-PCR	quantitative real-time polymerase chain reaction
IHC	Immunohistochemistry
H&E	Hematoxylin and eosin
DAB	3,3′-diaminobenzidine
KEGG	Kyoto Encyclopedia of Genes and Genomes
IFX	Infliximab
EMT	epithelial–mesenchymal transition
EndMT	endothelial-to-mesenchymal transition
*GDF15*	Growth Differentiation Factor 15
*MIF*	macrophage migration inhibitory factor
*TGFBR2*	TGF-β receptor type 2
*CXCR4*	C-X-C chemokine receptor type 4
*CD74*	cluster of differentiation 74
ChIP-seq	chromatin immunoprecipitation sequencing
CUT&Tag	Cleavage Under Targets and Tagmentation
PDOs	patient-derived organoids
