# Supplementary material for: Multi-Omics and Machine Learning-Based Characterization of the Lactylation Microenvironment and Biomarker Identification in Crohn’s Disease Intestinal Fibrosis
Source: Int J Mol Sci. 2026 Jul 17;27(14):6343. doi: 10.3390/ijms27146343 (PMC13410088; doi:10.3390/ijms27146343)
Supplement: Supplementary file 1 [file ijms-27-06343-s001.zip › Supplementary Table S1.pdf]

Accession number	Platform	Data type	Original dataset design / tissue source	Samples analyzed in this study	Inclusion and exclusion criteria applied in this study	Role in this study
GSE282122	GPL24676	scRNA-seq	Intestinal biopsy samples from IBD patients undergoing adalimumab treatment, including CD, UC, longitudinal treatment-related samples, and healthy controls. The original dataset is an adalimumab-treatment single-cell atlas of CD and UC.	28 CD inflamed lesion samples / 12 Healthy controls	UC samples, post-treatment samples, and other samples not matching the study objective were excluded. Only CD inflamed lesion samples and Healthy controls were retained.	Primary scRNA-seq analysis to define the intestinal cellular landscape, calculate lactylation scores, identify hyper-lactylated enterocyte subpopulations, and support downstream hdWGCNA, pseudotime, and CellChat analyses.
GSE186582	GPL570	Bulk transcriptome	Ileal samples collected from inflamed ileum (M0I), ileal margin (M0M), post-operative 6-month endoscopy (M6), and non-IBD ileal controls. The original dataset contains 520 CD-related samples and 25 non-IBD controls.	196 CD inflamed ileal mucosal samples (M0I) / 25 non-IBD controls	Only M0I inflamed ileal mucosal samples and non-IBD controls were included. M0M, M6, and other samples not used for the diagnostic training purpose were excluded.	Machine learning training set for initial screening of lactylation-associated candidate genes and construction of diagnostic models.
GSE66407	GPL19833	Bulk transcriptome	Gut/intestinal biopsy samples from patients with Crohn's disease, ulcerative colitis, and healthy/non-IBD controls.	47 CD samples / 99 controls	CD and control intestinal biopsy samples were retained. UC and other non-target samples were excluded from the present analysis.	Independent testing and external validation cohort for evaluating diagnostic efficacy of machine-learning models and core biomarkers CALD1 and CALM1.
GSE59071	GPL6244	Bulk transcriptome	Colonic mucosal biopsies obtained at endoscopy from UC patients, CD patients, and controls. The original GEO design includes 97 UC, 8 CD, and 11 controls.	8 CD samples / 11 controls	Only CD and control colonic mucosal biopsy samples were retained. UC samples were excluded.	Auxiliary clinical diagnostic model set supporting construction and validation of the CALD1/CALM1-based diagnostic nomogram.
GSE16879	GPL570	Bulk transcriptome	Mucosal biopsies from IBD patients before and 4–6 weeks after the first infliximab treatment, with response classified by endoscopic and histological findings; normal mucosal samples from controls were also included.	73 CD samples (37 pre-treatment and 36 post-IFX samples) / 12 controls	UC samples were excluded. CD-related pre-treatment and post-treatment samples, including responders and non-responders, and normal controls were retained for treatment-response analysis.	Anti-TNF-α/IFX treatment-response analysis to evaluate whether CALD1 and CALM1, especially CALM1, predict therapeutic response.
GSE228360	GPL24676	Spatial transcriptomics	Spatial sequencing data from intestinal tissue sections of CD patients, used to analyze spatial	3 CD spatial tissue sections	CD intestinal tissue sections used for spatial mapping were retained. This	Spatial transcriptomic validation to map hyper-lactylated enterocytes and assess their spatial

Accession number	Platform	Data type	Original dataset design / tissue source	Samples analyzed in this study	Inclusion and exclusion criteria applied in this study	Role in this study
			organization of inflammatory CD intestinal tissue.		analysis focused on spatial localization of scRNA-seq-defined cell populations in CD lesions.	proximity to endothelial cells and macrophages.

Abbreviations: CD, Crohn's disease; UC, ulcerative colitis; IBD, inflammatory bowel disease; scRNA-seq, single-cell RNA sequencing; hdWGCNA, high-dimensional weighted gene co-expression network analysis; IFX, infliximab.
