# Supplementary material for: Multi-Omics and Machine Learning-Based Characterization of the Lactylation Microenvironment and Biomarker Identification in Crohn’s Disease Intestinal Fibrosis
Source: Int J Mol Sci. 2026 Jul 17;27(14):6343. doi: 10.3390/ijms27146343 (PMC13410088; doi:10.3390/ijms27146343)
Supplement: Supplementary file 1 [file ijms-27-06343-s001.zip › Supplementary Table S3.pdf]

| Score | Body weight loss | Stool consistency | Fecal blood / hematochezia |
|---|---|---|---|
| 0 | No weight loss | Normal stool | Negative |
| 1 | 1–5% weight loss | Slightly soft stool | Weakly positive occult blood |
| 2 | 5–10% weight loss | Loose stool | Occult blood positive |
| 3 | 10–20% weight loss | Very loose stool | Visible blood in stool |
| 4 | >20% weight loss | Diarrhea | Gross bleeding or blood around the anus |

Total DAI score = body weight loss score + stool consistency score + fecal blood score.The total score ranges from 0 to 12, with higher scores indicating more severe colitis activity.
