# Supplementary material for: Multi-Omics and Machine Learning-Based Characterization of the Lactylation Microenvironment and Biomarker Identification in Crohn’s Disease Intestinal Fibrosis
Source: Int J Mol Sci. 2026 Jul 17;27(14):6343. doi: 10.3390/ijms27146343 (PMC13410088; doi:10.3390/ijms27146343)
Supplement: Supplementary file 1 [file ijms-27-06343-s001.zip › Supplementary Table S4.pdf]

Table S4 Primers used in qRT-qPCR experiments

Species	Name	Forward primer	Reverse primer
Mouse	CALD1	5′-CTGTCAGAGGACAAGAAGCCGT-3′	5′-GGAGACTACTGCTGCTTGGTGA-3′
	CALM1	5′-GCACCATCACAACCAAGGAACTG-3′	5′-ACTCTGGGAAGTCAATGGTGCC-3′
Human	CALD1	5′-CTGTTCCTGCTGAAGGTGTACG-3′	5′-CCTACCTTCAAGCCAGCAGTTTC-3′
	CALM1	5′-CCAACAGAAGCTGAATTGCAGGA-3′	5′-CAAAGACTCGGAATGCCTCACG-3′
