# Supplementary material for: Multi-Omics and Machine Learning-Based Characterization of the Lactylation Microenvironment and Biomarker Identification in Crohn’s Disease Intestinal Fibrosis
Source: Int J Mol Sci. 2026 Jul 17;27(14):6343. doi: 10.3390/ijms27146343 (PMC13410088; doi:10.3390/ijms27146343)
Supplement: Supplementary file 1 [file ijms-27-06343-s001.zip › Supplementary Table S5.pdf]

Parameter	0	1	2	3
Epithelial injury	Normal epithelium	Mild epithelial disruption or focal epithelial damage	Moderate epithelial erosion or partial epithelial loss	Severe epithelial destruction, extensive erosion, or ulceration
Inflammatory-cell infiltration	No obvious inflammatory infiltration	Mild inflammatory infiltration, mainly limited to mucosa	Moderate infiltration extending within mucosa/submucosa	Severe dense infiltration, extensive or transmural inflammatory involvement
Crypt architecture	Normal crypt structure	Mild crypt distortion or focal crypt irregularity	Moderate crypt loss or architectural distortion	Severe crypt destruction, marked crypt loss, or complete architectural disruption
Mucosal edema	No edema	Mild mucosal edema	Moderate mucosal/submucosal edema	Severe diffuse edema with marked tissue expansion
