# Supplementary material for: Multi-Omics and Machine Learning-Based Characterization of the Lactylation Microenvironment and Biomarker Identification in Crohn’s Disease Intestinal Fibrosis
Source: Int J Mol Sci. 2026 Jul 17;27(14):6343. doi: 10.3390/ijms27146343 (PMC13410088; doi:10.3390/ijms27146343)
Supplement: Supplementary file 1 [file ijms-27-06343-s001.zip › Supplementary_Material.pdf]

Supplementary Material

Supplementary Figures

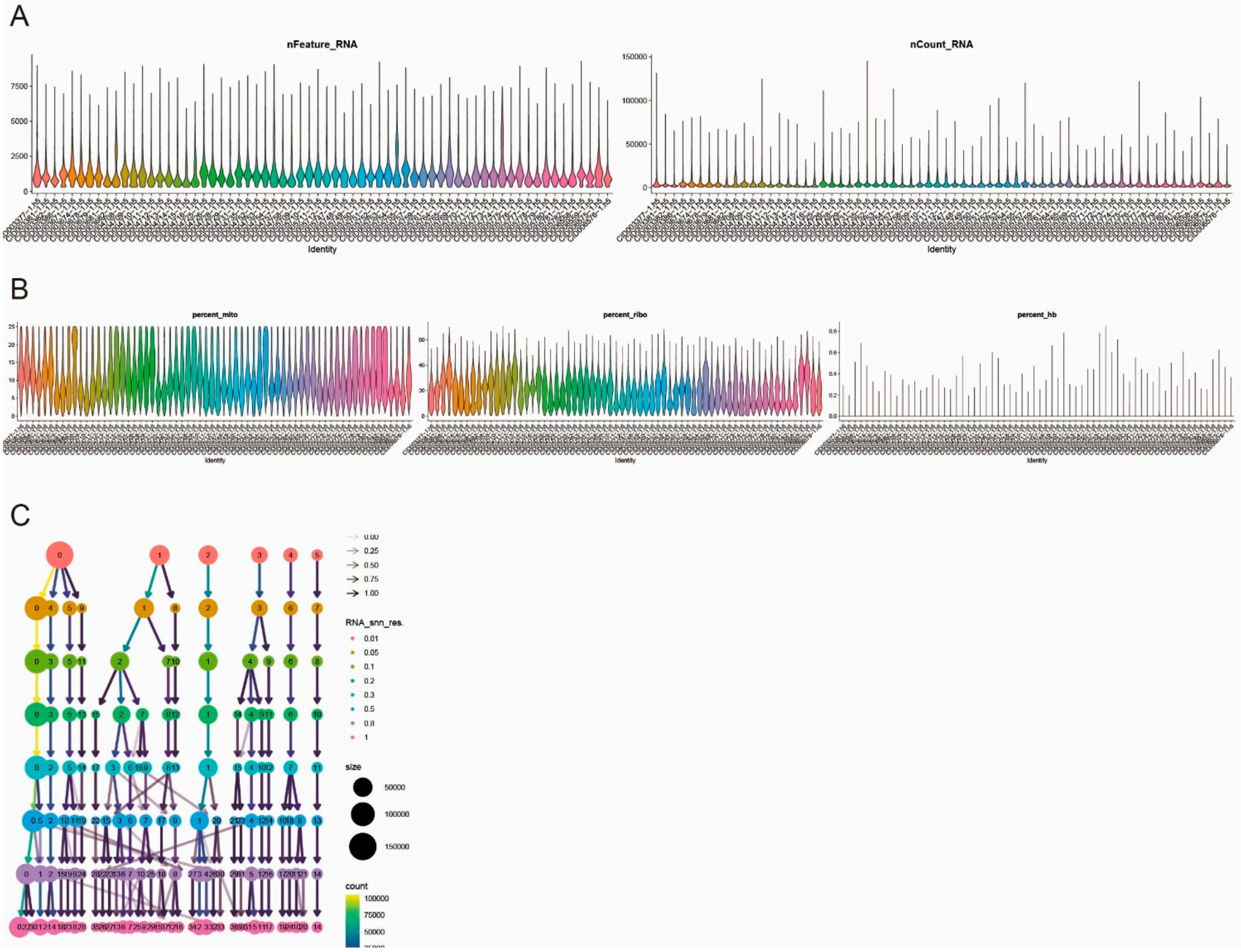

Supplementary Figure S1. Evaluation of Quality Control Metrics and Clustering Resolution Selection for Single-Cell Transcriptome Data.(A) Violin plots showing the distribution of the number of genes detected per cell (nFeature_RNA, left) and total UMI counts (nCount_RNA, right) across all samples, used to evaluate sequencing depth and gene capture efficiency.(B) Violin plots of key quality control metrics, displaying the distribution of mitochondrial gene percentage (percent_mito, left), ribosomal gene percentage (percent_ribo, middle), and hemoglobin gene percentage (percent_hb, right) in each sample.(C) Clustree clustering tree analysis. Showing the evolution and branching of cell clusters from resolution 0.01 to 1.0, which was used to guide the selection of an appropriate clustering resolution for downstream analysis.

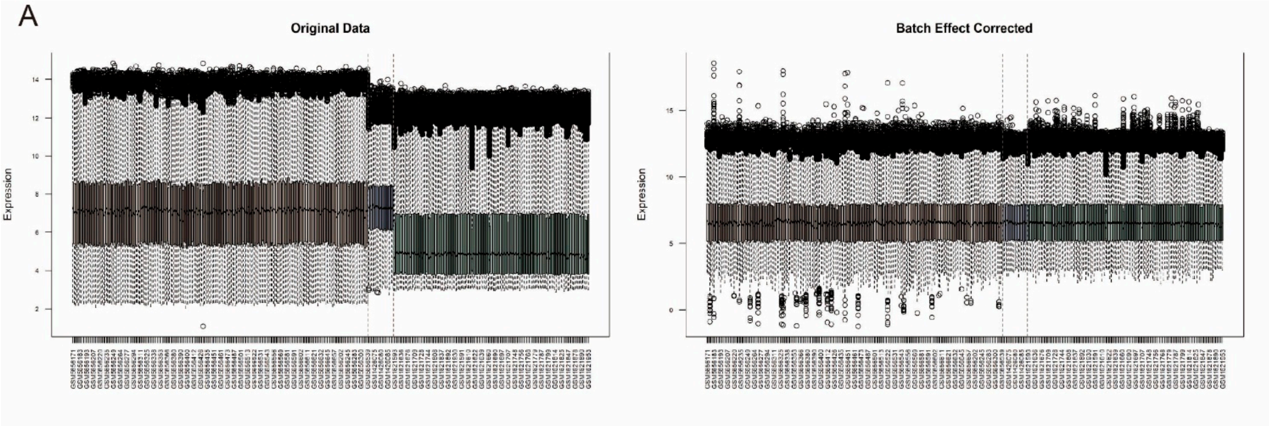

Supplementary Figure S2. Data Preprocessing and Batch Effect Removal. (A)Box plots show the gene expression distribution of the merged dataset before batch effect removal (left panel, Original Data) and after batch effect correction (right panel, Batch Effect Corrected). The results indicate that the data distribution among samples becomes consistent after correction, with systematic bias eliminated, making it suitable for subsequent analysis.

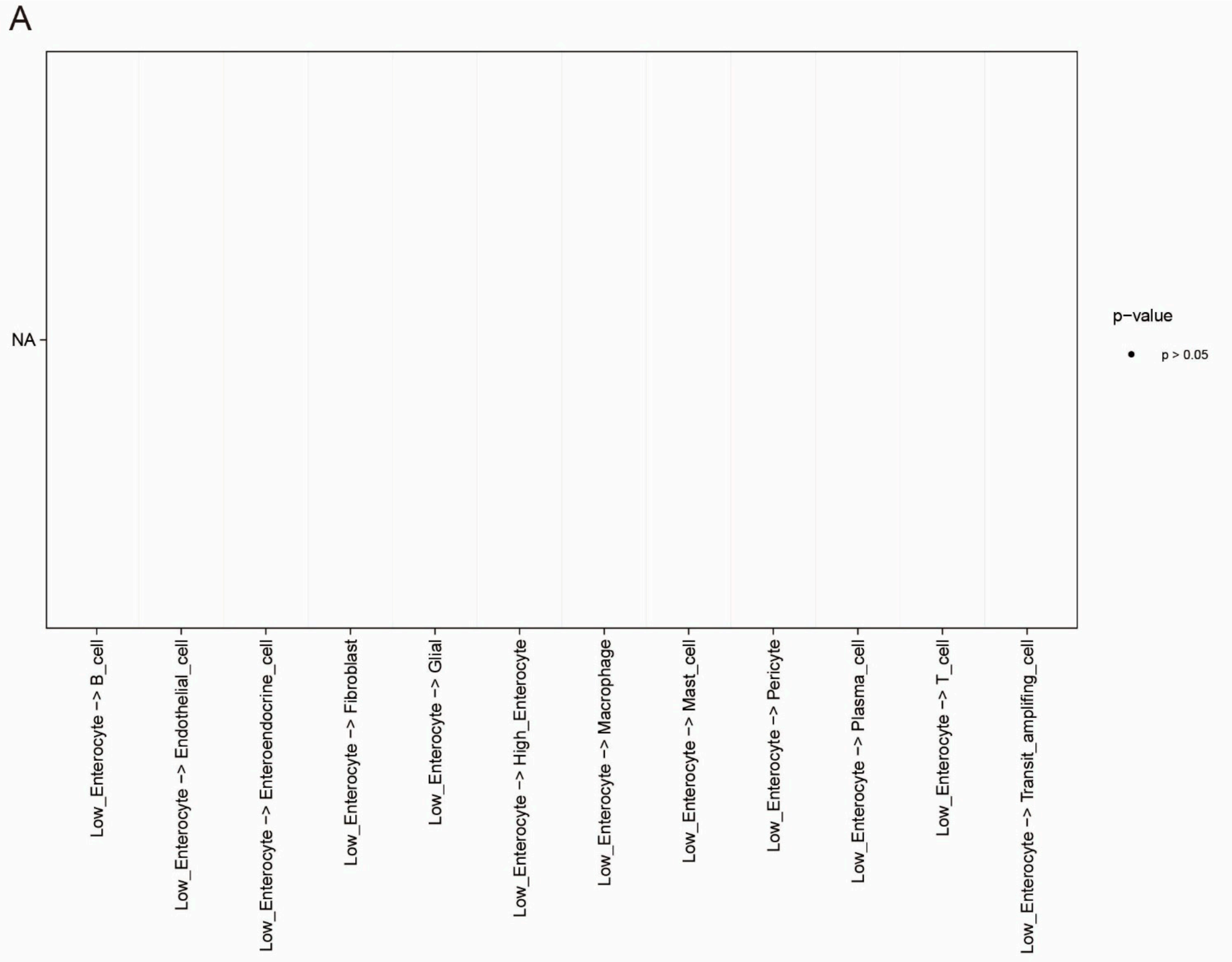

Supplementary Figure S3. Analysis of Intercellular Communication Network in Intestinal Epithelial Cells.(A) Bubble plot of ligand-receptor interactions.
